# Decomposing logophoric pronouns: a presuppositional account of logophoric dependencies

**DOI:** 10.1007/s11050-025-09242-x

**Published:** 2025-10-30

**Authors:** Itai Bassi, Imke Driemel, Abigail Anne Bimpeh, Silvia Silleresi, Johnson Fọlọrunṣọ Ilọri, Anastasia Nuworsu, Gerald Okey Nweya

**Affiliations:** 1 Leibniz-ZAS, Pariser Straße 1, Berlin, 10719 Germany; 2 University of York, Heslington, York, YO105DD UK; 3 Humboldt-Universität zu Berlin, Unter den Linden 6, Berlin, 10099 Germany; 4 University of Milano-Bicocca, Piazza dell’Ateneo Nuovo 1, Milan, 20126 Italy; 5 University of Lagos, University Road Lagos Mainland Akoka, Lagos, Nigeria; 6 Ho Technical University, HFRC+M4R Poly Rd, Ho, Ghana; 7 University of Ibadan, Ibadan 200001, Oyo, Nigeria

**Keywords:** Logophors, Strict/sloppy readings, Binding, Presupposition, Ewe, Yoruba, Igbo, Focus, de se, Attitude ascriptions

## Abstract

Logophoric pronouns in West African languages occur in attitude environments and are anaphorically linked to an attitude holder in a superordinate clause. This has motivated theorists to treat logophoric pronouns semantically as an obligatorily bound variable (bound from the edge of an embedded complement clause). Culy (Linguistics 32:1055–1094, 1994) and Bimpeh and Sode (in Proceedings of TripleA, vol. 6 pp. 1–16, 2021), however, point out that logophoric pronouns in Ewe do not behave like obligatorily bound variables, allowing both sloppy (bound) and strict (non-bound) readings in focus contexts involving ‘only’ and ellipsis. We strengthen this line of criticism by providing novel cross-linguistic data that indicate that logophoric pronouns in Ewe, Igbo and Yoruba support strict readings. We offer an alternative formal account to existing approaches that builds on Bimpeh et al. (in Proceedings of the 40th WCCFL, pp. 1–10, 2024) and can capture both strict and sloppy interpretations, while preserving the requirement that a logophoric pronoun be anaphoric to an attitude holder. The main novelty involves decomposition of logophoric pronouns into two syntactic components at LF—a variable that can in principle be free and refer strictly, and a semantic presuppositional feature log that can be ignored in ellipsis and focus sites, following similar ideas in the literature on pronominal features (Sauerland in Proceedings of SALT 23, pp. 156–173, 2013). Our analysis implies that in terms of their syntactic and semantic make up, logophors are essentially no different from other pronouns, consisting of a referential index plus semantic features.

**Supplementary Information:**

The online version contains supplementary material available at 10.1007/s11050-025-09242-x.

## Introduction

Logophoric pronouns in some West African languages are special anaphoric elements that typically occur in attitude contexts and must refer back to the attitude holder (Clements 1975). In Ewe, for example, the logophoric pronoun (henceforth LogP) *yè* normally appears in attitude ascriptions like in (1). For convenience, the relationship between LogP and its antecedent is represented with indexation.^1^


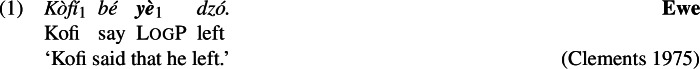


Ewe’s LogP is restricted to this kind of environment; in particular, it cannot be used in simple unembedded sentences to refer to an antecedent introduced in the preceding discourse (or made contextually salient otherwise). For instance, if Afi is the topic of conversation and we mention her having been at the party, then (2a) cannot be used as a follow-up; in such cases, the ordinary third person pronoun (henceforth OrdP) *é* must be used (2b).


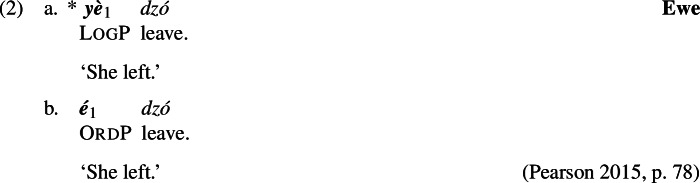


Clements (1975), one of the first to systematically study the phenomenon, thus characterizes Ewe’s LogP *yè* as an item “used exclusively to designate the person whose speech, thoughts, feelings or general state of consciousness are reported.” In the literature since the 70s, the term “logophoricity” has been applied to describe elements with a similar function of encoding sensitivity to the attitude of some perspective-holder (see Hagège 1974; Charnavel 2020 for French; Koopman and Sportiche 1989 for Abe; Nikitina 2012 for Wan; Kaiser 2018 for Finnish; Park 2018 for Korean; Sundaresan 2018 for Tamil; Kiemtoré 2022 for Jula; Newkirk 2019 for Ibibio; Schlenker 1999 for Amharic; and Sells 1987; Culy 1994; Stirling 1994; Güldemann 2003; Deal 2020 for cross-linguistic studies).

How is the dependency between LogP and its antecedent encoded in the grammar? This paper is an attempt to advance toward an answer to this question, from the angle of the well-known strict-sloppy ambiguity of pronominal reference: our discussion and analysis will gear toward explaining why LogPs allow strict readings in ellipsis and focus contexts (cf. Culy 1994; Bimpeh and Sode 2021).

As an overview, we provide new data from three languages—Ewe, Yoruba and Igbo—that confirm that LogPs in these languages are ambiguous between sloppy and strict interpretations (our work also provides the first cross-linguistic study on *de se* readings of LogPs that includes mistaken identity scenarios across several attitude predicates). We will discuss why the existence of the strict reading is problematic for current approaches (von Stechow 2003; Pearson 2015, a.o.), and will account for the problematic generalizations with a novel theory of the syntax-semantics interface of LogPs. The main novelty is a decomposition of logophoric pronouns into two syntactic components at Logical Form (LF)—a variable and a semantic feature log that induces a presupposition, like other pronominal features.

The paper is organized as follows. Section 2 provides general background on the basic distribution and interpretation of logophors in Ewe, Yoruba and Igbo. Section 3 presents our findings on strict and sloppy interpretations, and shows why they require modification of the existing accounts. Section 4 presents our new proposal for a syntax-semantics of LogPs that can capture strict readings, including hitherto undescribed flavours of strict readings. Section 5 expands the proposal and accounts for long-distance LogP dependencies and for the relationship between LogP and the first person pronoun in the languages of interest.

## Logophors and their distribution and interpretation

We focus on three West African languages that have been observed to display logophoric pronouns: the Kwa language Ewe, and two Benue-Congo languages: Yoruba and Igbo. Detailed language profiles can be found in the Supplementary Material accompanying this paper. In Sect. 2.1, we present the main distributional pattern of LogPs in the languages under investigation, while Sect. 2.2 is devoted to a discussion of the *de se*-*de re* distinction in connection to logophors.

Unless indicated otherwise, all data in this paper come from original fieldwork. We elicited data from three Ewe speakers (two Anlo and one Ewedome), two Yoruba speakers and four Igbo speakers. All data was elicited via multiple Zoom sessions with each speaker, transcribed live by the experimenters and double-checked by the speakers. The elicitation language was English. Speakers’ spontaneous comments on the reasoning behind their responses were also noted.^2^

### Obligatory co-reference with the attitude Holder

In (3), we present data for Ewe’s LogP *yè* embedded under the attitude predicates ‘think’, ‘say’, ‘want’, and ‘hope’.^3^ As indicated by the indexation, *yè* must co-refer with the attitude holder in the matrix clause; it cannot refer to some other contextually salient individual.


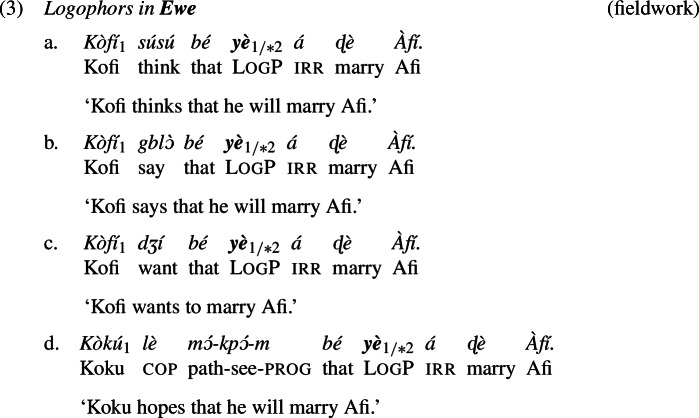


Yoruba and Igbo have LogPs that exhibit comparable distributional properties to those of Ewe (Manfredi 1987; Hyman and Comrie 1981; Adésolá 2005; Lawal 2006). Examples (4) and (5) illustrate.^4^


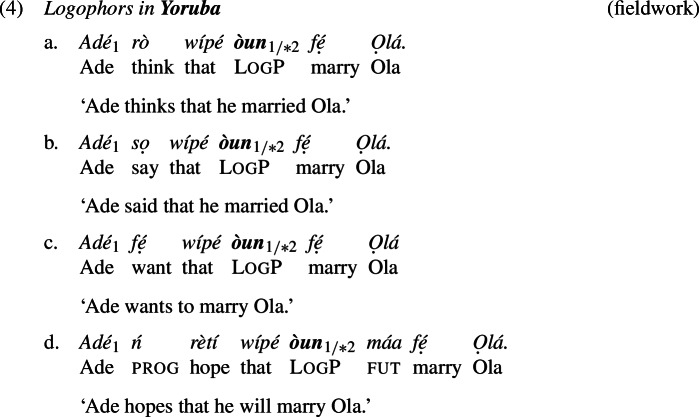



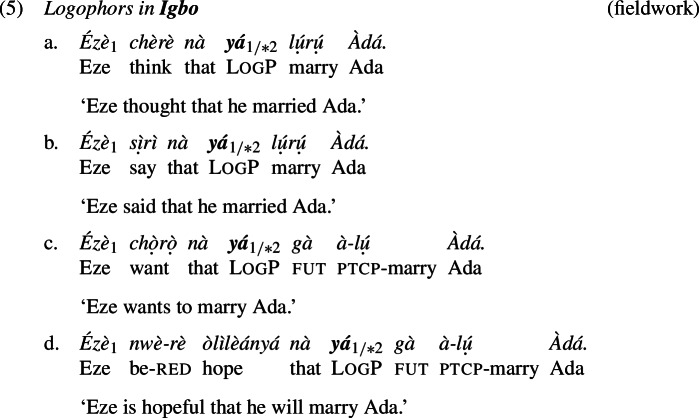
All three languages show identical co-reference patterns. The results of our elicitation sessions confirm previous reports in the literature. In the next section, we will report on another trademark property of logophors: obligatory *de se* readings.

### The *de se*-*de re* distinction

For the elicitation of the data in this section, we used a binary acceptability judgment task designed with joint presentation for two target sentences (one with LogP and one OrdP) to be judged against *de re* contexts: speakers were asked to express their acceptability judgments on both target sentences, but they were free to accept as felicitous both sentences, one sentence or none. Additionally, consultants were asked to judge the same target sentences against equivalent *de se* scenarios (see Sect. 2 in Supplementary Material) to confirm our methodology and the data in Sect. 2.1.

#### *de se*-only interpretation of logophors

It falls beyond the scope of this paper to provide a complete overview of the literature on LogPs.^5^ But one property of LogPs that will be integrated into our analysis deserves elaboraton, and that is the *de se* reading of LogPs. The so-called *de se*-*de re* distinction of pronominal reference in attitude contexts has to do with whether the attitude holder recognizes themselves as the real referent of the pronoun (Lewis 1979, Chierchia 1989, Kaplan 1989, Schlenker 1999, Pearson 2015, Park 2018, Patel-Grosz 2020, a.o.). Recently, Bimpeh et al. (2024) found evidence that LogPs in Ewe, Yoruba and Igbo must meet this “self-awareness” requirement. Specifically, Bimpeh et al. (2024) showed that LogPs (but not OrdPs) are infelicitous in “mistaken identity” scenarios as in (6), in which Donald Duck is referring to someone who, unbeknownst to Donald, is actually him.


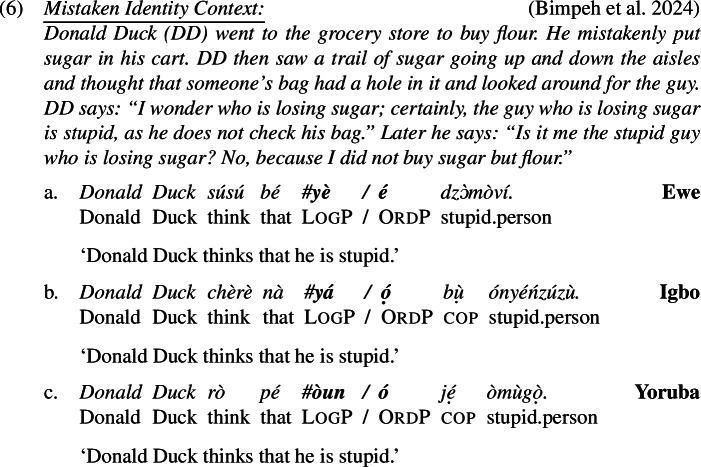


As with ‘think’ (6), logophors embedded under ‘say’ and ‘hope’ can only be read *de se*, highly suggested by the infelicity in a mistaken identity scenario in (7) and (8).


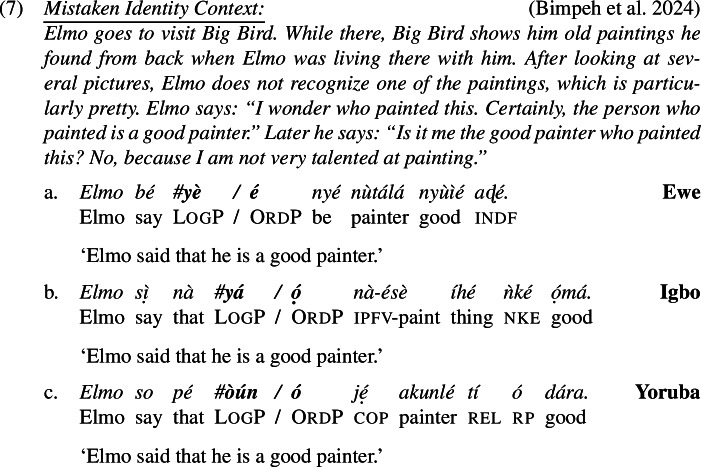



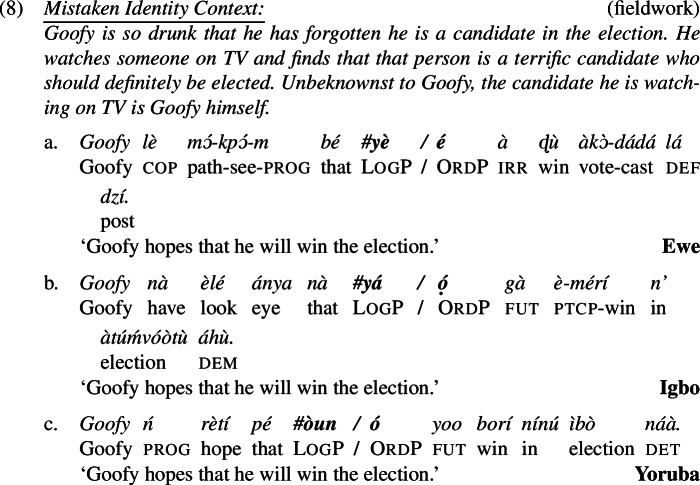


We take it then that LogPs in these languages have a requirement for *de se* readings: LogP can only refer to the attitude holder’s “recognized self”.

#### A note on methodology and conflicting generalizations

Our claim that LogPs only have *de se* readings is controversial. While it converges with the results in Bimpeh (2019) for Ewe, as well as with Adésolá (2005) and Anand (2006) for Yoruba (we are not aware of a previous discussion of *de se*-*de re* with respect to Igbo), Pearson (2015) by contrast reports that LogP in Ewe also supports a *de re* reading, as most of the speakers in her study judged LogP as felicitous and true in mistaken identity scenarios (see also O’Neill 2015; Satık 2021). These findings were partly confirmed by a quantitative questionnaire study on *de se*-*de re* readings by Bimpeh (2023) with 20 Ewe speakers. While 62.5% responses indicated that LogP is infelicitous in mistaken identity contexts, 37.5% responses revealed that LogP was accepted. This indeed suggests that LogP at least sometimes seems to receive a *de re* reading. The questionnaire study in Bimpeh (2023), however, revealed several surprising results: most importantly, OrdP was rejected by participants over half of the times (OrdP was judged infelicitous in mistaken identity scenarios 65.7% of the time). This outcome is unexpected under every account mentioned so far. Bimpeh (2023, p. 127) concludes that “a possible explanation [for the discrepancy in results between Bimpeh (2023) and Bimpeh (2019)—our addition] is that participants were mostly confused since they are not used to such scenarios”, which is possibly why LogP and the baseline OrdP version were both rejected over 50% of the time. The difference between one-on-one elicitation (the current study and Bimpeh 2019) and questionnaires (Bimpeh 2023) is that with the former, consultants can more comfortably be familiarized with the rather unusual mistaken identity contexts, and ask for clarification if necessary. Consequently, the one-on-one elicitation method used in Bimpeh (2019) achieved consistent results indicating exclusively *de se* interpretation of LogPs, which align with our findings.

It is important to try to clarify the nature of the disagreement, and to this end we now comment about the methodology we used to obtain our results in (6)-(8) and explain how it differs from studies that arrived at conflicting conclusions to ours. Our technique to elicit *de re* readings is different from previous work on two points. First, as is clear from (6)-(8), and following Bimpeh’s (2023) idea, we let our speakers judge not just sentences with LogP but also a minimally different variant with OrdP. This gave speakers the opportunity to create a baseline, and it also allowed us to verify that speakers understood the context (they were asked to express their acceptability judgments on both versions, and were free to accept both sentences, one sentence or none). We think this is highly relevant, as mistaken identity contexts are rather difficult to take in (especially for non-linguists/semanticists). With the exception of Bimpeh (2023), previous work (Pearson 2015; O’Neill 2015; Satık 2020, 2021) has not tested the OrdP version in mistaken-identity scenarios to establish a baseline. The fact that our speakers all accepted the OrdP version but rejected the LogP version suggests that only the former is compatible with a *de re* reference.

Second, most of our mistaken-identity scenarios made sure that the target sentences are false on a *de se* reading. In the mistaken identity context in (6), for example, it is explicitely mentioned that Donald Duck does not self-ascribe stupidity. (“Is it me the stupid guy who is losing sugar? No, because I did not buy sugar but flour.”) No previous work we are aware of (Pearson 2015; O’Neill 2015; Satık 2020, 2021; Bimpeh 2023) made it unambiguously clear in the description of the context that a *de se* interpretation is false. We believe it is important do to so, in order to make sure that upon judging the LogP sentence, speakers do not apply some charity principle and mentally modify the mistaken-identity context ever so slightly so as to make it possible for the sentence to be true on a *de se* reading. Whenever our speakers were asked if the sentence is true or false against a mistaken-identity context that made it sufficiently clear that a *de se* interpretation is false, our speakers indicated to us that the sentence is false.^6^ For more discussion on the methodology, see the Supplementary Material.

## Strict readings of logophors

### The simplex binding account of logophors and a problematic prediction

In the formal semantic literature on LogPs, it has become standard to capture their basic distributional facts—namely, the requirement for (*de se*) coreference with the attitude holder—by assuming that LogPs are simplex bound variables, bound from the left periphery of complement clauses. This is the view taken for example by Schlenker (2003), von Stechow (2004), Heim (2005), and Pearson (2015). We will call this the Simplex Binding approach to LogPs. Let us briefly go over how this approach works, as it will later be criticized based on the availability of strict readings of LogPs. We use the account in Pearson (2015) to illustrate the approach.^7^

Pearson (2015), following von Stechow (2003), assumes that LogP is like a standard pronoun in being interpreted as a bare variable (via an assignment function as usual), but it comes with a syntactic feature log whose purpose is to make sure that that variable ends up being bound by a *λ*-operator at the edge of an embedded clause (technically by feature “checking” between [log] and the matching *λ*). To illustrate, the LF representation of *Kofi says that* LogP *will marry Afi* is in (9a), where [log] enforces index matching between the variable and the binder at the edge of the CP (the boldfaced *λ* operator). This syntax is coupled with a semantics that assigns the embedded clause a property meaning (type 〈e,st〉), and an appropriate meaning for attitude predicates like *say* that involves quantification over *centered worlds* (Lewis 1979); see (9b).


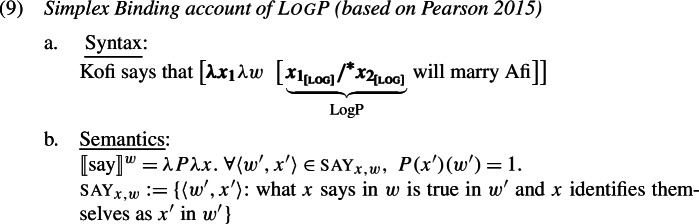
In (9a), the fact that [log] requires $x_{1}$ to be bound by $\lambda x_{1}$ makes sure that LogP ends up referring to the attitude holder’s recognized self (the “Logophoric Center”), and this yields obligatory *de se* coreference with the attitude holder. The paraphrase of the resulting meaning is in (10).


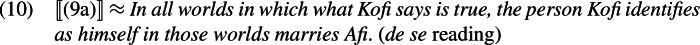


The Simplex Binding approach to LogPs makes a prediction about the possible readings of LogP with respect to the strict-sloppy ambiguity: it predicts that LogP cannot have a strict reading in sentences with *only* and in ellipsis. The prediction comes about on standard assumptions about semantic interpretation, namely that bound-variable representations (*λ*-binding at LF) translate to sloppy readings in focus and ellipsis environments (Ross 1967; Partee 1973; Sag 1976; Williams 1977; Reinhart 1983; Heim and Kratzer 1998).

The prediction, however, is not borne out: as we show below, LogPs in the languages we investigate do admit strict readings (alongside sloppy readings, as expected). The problem has already been highlighted by Culy (1994) and Bimpeh and Sode (2021) for Ewe, and below we provide comparable cross-linguistic data from Ewe, Yoruba and Igbo, confirming this conclusion.

### Strict readings across Ewe, Yoruba, and Igbo

Examples (11)-(12) show that in environments involving *only* (association with focus), there is both sloppy and, crucially, strict readings for LogP. We used a binary acceptability judgment task designed with joint presentation for both strict and sloppy interpretations of the target sentence: speakers were asked to express their acceptability judgments on both paraphrases (one *strict* and one *sloppy*), but they were free to accept as felicitous both sentences, one sentence or none. In each language, the paraphrases were accepted as felicitous interpretations of the target sentence.


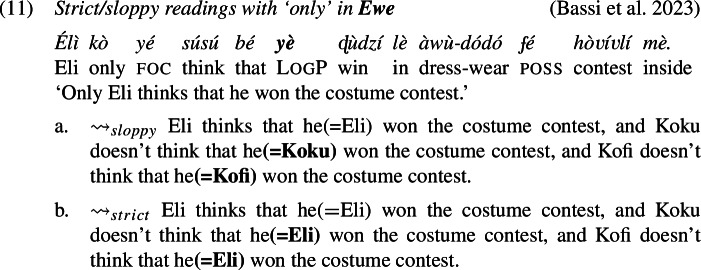



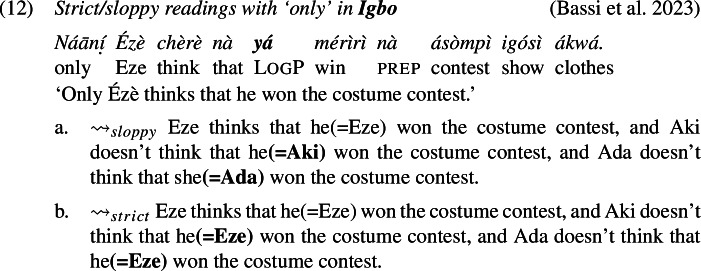



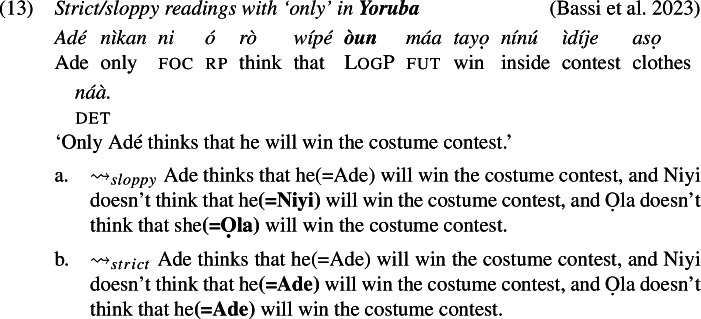


Other relevant environments for testing the prediction are ellipsis configurations. To investigate strict and sloppy identity in ellipsis, we did not make use of VP ellipsis, as this type of ellipsis is not found in the relevant languages. Argument ellipsis is also not an option. As shown in (14), a structure set up to test for strict/sloppy identiy involving  ‘say’ in Ewe requires an overt pronoun.


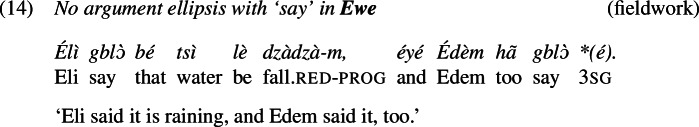


To our surprise, we also encountered difficulties when testing for stripping configurations, at least for the ‘say’/‘think’-type verbs. For example, not all of our consultants accepted (15).


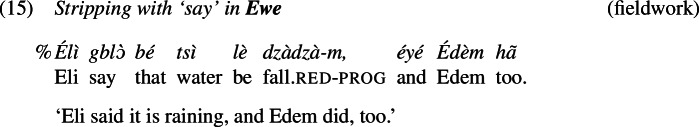


We did not encounter such difficulties with the verb ‘hope’, that is, stripping was accepted by all of our consultants for each language. Thus, we demonstrate the availability of strict (and sloppy) readings with logophors embedded under ‘hope’, which is shown for Ewe in (16), for Igbo in (17), and for Yoruba in (18).


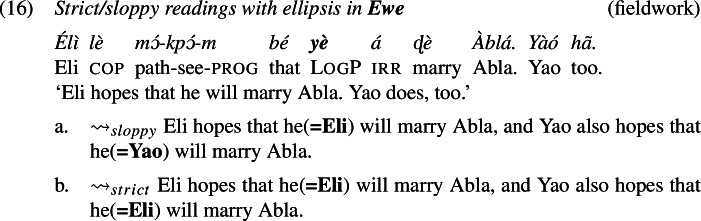



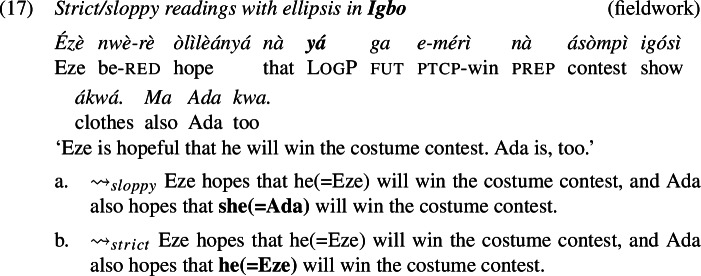



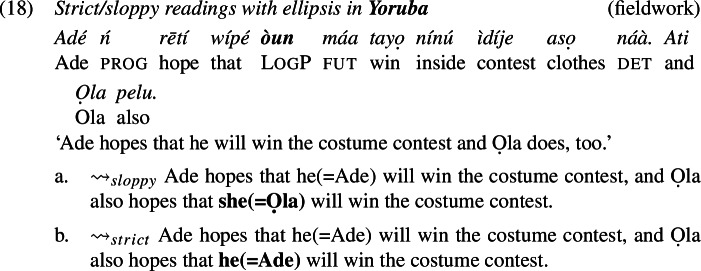


### The analytical problem

In the previous section, we demonstrated the robust availability of strict readings of logophors in focus and ellipsis constructions. The present section lays out the problem this raises for Simplex Binding.

Consider first ellipsis. If ellipsis requires LF/semantic identity (“Parallelism”) between the elided phrase and an antecedent phrase (Keenan 1971; Sag 1976; Williams 1977; Rooth 1992a; Tancredi 1992; Fiengo and May 1994; Takahashi and Fox 2005; Merchant 2019, a.o.), then the Ewe sentence in (16), for instance, should be schematically analyzed as in (19) (grey material indicates ellipsis).


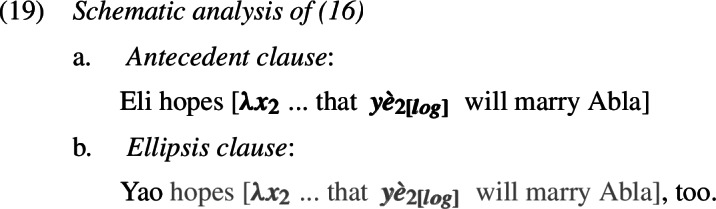
Because of the Simplex Binding assumption, the two LogPs—the overt and the elided one—must be bound by the edge of their respective embedded clauses. This produces a sloppy reading: Yao hopes (*de se*) that he himself will marry Abla. Simplex Binding permits no other representation that both respects the identity condition on ellipsis and can produce a strict reading.

The cases with *only*, for instance in (11), present a similar binding problem. To show this, we need to be specific about the analysis of *only* in Ewe, Yoruba and Igbo, but any sensible analysis will do. Concretely, we assume—also for the purpose of preparing the grounds for our proposal later—that these sentences involve the computation of focus alternatives, similar to what is widely assumed for English *only* (e.g., Rooth 1992b). A schematic analysis of (11) is given in (20). The subject *Eli* is marked with the feature Foc(us), which generates alternative structures created by substituting *Eli* with some (relevant) individual. *Only* negates the alternatives.


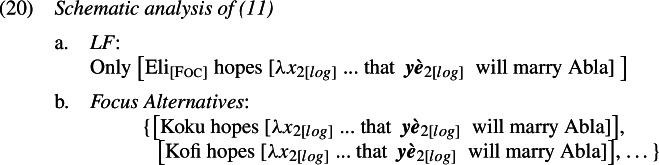
With these alternatives, the sloppy reading is accounted for: because *yè* is *λ*-bound, in each of the alternatives the position of *yè* is co-valued (*de se*) with the relevant alternative to Eli. The strict reading, however, requires a different representation, one that appears to be at odds with Simplex Binding, where the value of *yè* would remain constant across all alternatives and pick out Eli.

Something, then, must be changed in the theory. The dilemma we are faced with can be stated as in (21).


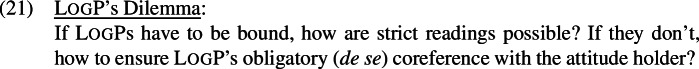


In the next section, we turn to our perspective on the dilemma. We will reject Simplex Binding and propose a decompositional analysis of LogPs that captures the (*de se*) co-reference requirement of LogPs with a richer structure for LogPs than so far assumed, one that gives room for strict readings to emerge.

## Proposal: a decompositional approach to logophoric dependencies

To preview, we propose that the logophoric pronoun (in Ewe, Yoruba and Igbo) underlyingly consists of two syntactic pieces: roughly, . *pro*_*i*_ is a variable over individuals with no binding requirements: it may be free or *λ*-bound from above. log is a presuppositional pronominal feature that roughly equates the reference of *pro*_*i*_ with the (*self*-counterpart of the) attitude holder, and this produces the *de se* coreference property of LogPs. Our proposal also crucially builds on recent ideas in the literature to account for the exceptional behavior of pronominal features in focus and ellipsis environments: their ability to be deactivated when computing focus alternatives and ellipsis identity (e.g., Jacobson 2012; Sauerland 2013). We will show that strict readings of LogPs are possible if log’s featural contribution can be suspended in essentially the same way.

If our analysis is correct, then LogPs are no different from other pronouns at LF in terms of their syntacto-semantic makeup. They contain a variable part and a semantic feature, just like, e.g., the English pronoun *her*_*i*_ consists of a variable that fixes the reference of the pronoun plus a presuppositional gender feature.

We now turn to the details of the analysis.

### Background: fake *ϕ*-features in focus alternatives

A well-known observation in the literature on binding, dating at least as far as Ross (1967), is that pronominal *ϕ*-features (gender, number, person) have a special status in ellipsis and focus enviroments: their contribution can be ignored across alternatives (see also Hestvik 1995; Heim 2008, among many others). Cases of fake indexicals in focus, as in (22a), as well as fake gender as in (22b), exemplify this:


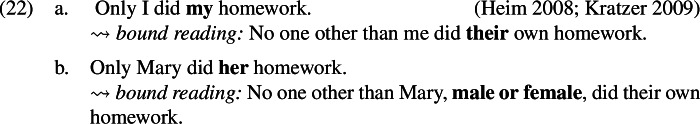


To account for such behavior, a prominent approach stipulates something like the following principle (Jacobson 2012; Sauerland 2013; McKillen 2016; Sudo and Spathas 2020; Bassi 2021; Bruening 2021).


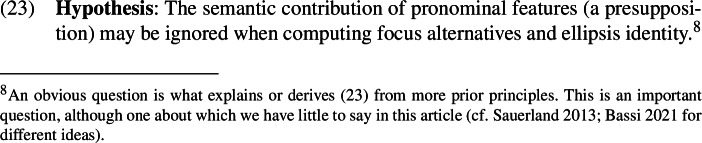


As previewed, our proposal to capture strict readings of LogPs relies crucially on this hypothesis. We will propose (Sect. 4.4) that the pronominal feature log contained in a LogP is one whose contribution can be ignored when computing focus alternatives, since it is subject to the hypothesis in (23).^8^

### Setting up a semantics of attitudes with counterparts

Before going further into our syntax-semantics of LogPs, we must first lay out some assumptions about the general framework. Any theory that incorporates an analysis of reference in attitude contexts must deal with well-known *de re* puzzles that have been discussed ever since the works of Quine (1956) and Kaplan (1968) (cf. also Lewis 1968; Percus and Sauerland 2003; Charlow and Sharvit 2014; Sauerland 2018, a.o.), and this is what we do in this section. Once we have a working system for modeling reference in (and binding into) attitude environments, we will show how our analysis of LogP neatly fits in, and how strict readings are accounted for as a virtue of the featural analysis of log.

This section may be regarded as somewhat of a long (though necessary) excursus. Readers who are not interested in the details of the assumed framework may wish to skip to Sect. 4.3.^9^

#### A Lewisian ontology, *de re* puzzles and counterparts

Our system is loosely based on Sauerland (2018) and Heim (2001), who make use of Lewis’s (1968) Counterpart ontology to build a compositional analysis of attitude ascriptions. On this ontology, individuals can only occupy one possible world, but an individual can have counterparts in other worlds—more on this below. A first stab at a Lewis-inspired LF for a sentence like *John thinks Mary is a spy* is given in (24). Attitude ascriptions involve quantification over centered worlds, which are pairs of a world and an individual. In our system, centered-world variables are represented in the structure and saturate argument slots in the denotation of verbal and nominal predicates (see, a.o., Percus 2000; von Fintel and Heim 2011). ‘$w_{x}$’ is shorthand for the world-individual pair <*w*,*x*>.^10^


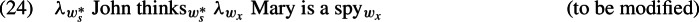


In (24), a variable binder over centered worlds is introduced at the edge of the CP and binds the centered-world variable on the predicate. Suitable denotations are given in (25a) and (25b) for simple predicates like *spy* and for attitude predicates like *think* (we notate with ‘s’ the semantic type of centered worlds). Throughout, we take individual-denoting phrases (proper names, pronouns) to be interpreted as simple type-*e*, actual-world individuals: 〚Mary〛 = the actual individual Mary.


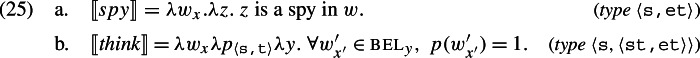


The domain of quantification for *think*, bel, is defined in (26). It is a set of centered worlds where the world coordinate *w* is as it usually is for belief predicates, and the individual coordinate *x* is a “recognized self” of the attitude holder.


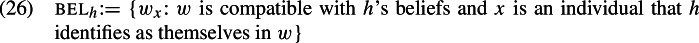


Put differently, the *x*-coordinate in (26) is an individual in *w* who, if *h* was put in *x*’s place, *h* would not experience any difference to what they take to actually be the case. We call *x* here a self-counterpart of *h* in *w*.

There are two (related) problems with the LF in (24) having to do with the occurrence of *Mary* in the embedded clause. First, since on our Lewis-adopted ontology individuals only occupy one possible world (no trans-world individuals), an interpretation like 〚(24$)]\!\!]= [\forall w_{x} \in \textsc{bel}_{John}, \ Mary \ is \ a \ spy\ in\ w]$ doesn’t make much sense; it entails that the predicate *spy*, evaluated in John’s belief worlds, applies to the actual Mary, who doesn’t inhabit those worlds, and this cannot be satisfied. We need our formalism to rather deliver that the embedded subject denotes not the actual Mary, but different counterparts of hers that live in John’s belief worlds. Second, this analysis is not equipped to account for the intuition that attitudes about an individual can be both true and false in so-called “double-vision scenarios”. These scenarios give rise to certain *de re* puzzles discussed since Quine (1956) and Kaplan (1968). The observation is that sometimes more than one way to specify counterparts of individuals is relevant for the interpretation of such sentences. Imagine that John has been acquainted with a certain Mary in two different ways: he sees her at the beach on a sunny day, and he also sees her trying to infiltrate into a military base at night. Crucially, John doesn’t know that the person at the beach and the infiltrator are in fact one and the same person. John suspects that the infiltrator is a spy, but has no reason to believe that the beach person is a spy. In this double-vision scenario, the sentence *John thinks Mary is a spy* is intuitively both true (by virtue of Mary being the night infiltrator) and false (by virtue of her being the beach person). Double-vision scenarios then point to the need for having a way to semantically encode how exactly attitude holders are acquainted with individuals.

We amend both of these problems, following Heim (2001) and Sauerland (2018), by enriching our representations to include Counterpart functions. Individuals indeed occupy only one possible world, but can be systematically linked to their counterparts in other worlds. The exact way they do so depends on some suitable Acquaintance function. The Counterpart technology amounts to a device that maps the evaluation-world individual Mary in (24) into a certain counterpart of Mary that lives in *w*.

Concretely, we postulate silent counterpart pronouns, $\boldsymbol{C^{f^{i}}_{x}}$, which can attach to individual-denoting phrases like *Mary*. An amended LF is in (27).


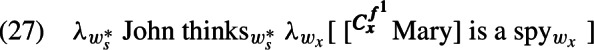


Informally, here is how counterpart pronouns work. They consist of two parameters: an indexed acquaintance function $f^{i}$, whose content we assume is supplied contextually (by an assignment function), and an individual “pivot” *x*, which is bound by an attitude holder and is therefore the individual’s *self*-counterpart in the relevant worlds. $f^{i}$ encodes a specific description, say ‘the woman seen at the beach’, by which actual-John is acquainted with actual-Mary. ‘$\boldsymbol{C^{f^{i}}_{x}}$(Mary)’ is to be read as *the f*^*i*^*-counterpart of Mary in the world of* *x*. So, ‘$\boldsymbol{C^{f^{2}}_{x}}$(Mary)’, for example, could be a counterpart of Mary in the world of *x* as she is described in the mind of *x* as “the woman I saw at the beach”; (27) would thus be true iff John thinks that that woman is a spy.

The Counterpart technology—or its parallels in other systems, notably Concept Generators (Percus and Sauerland 2003; Charlow and Sharvit 2014, a.o.)—allows us to provide an account for double-vision scenarios. A sentence like *John thinks Mary is a spy* uttered in a context where John is acquainted with Mary through two different descriptions as illustrated above could be represented either as in (28a) or (28b)—and one of them could be true while the other false. The difference is located in the different acquaintance functions, which determine different individuals as counterparts of the same actual Mary.


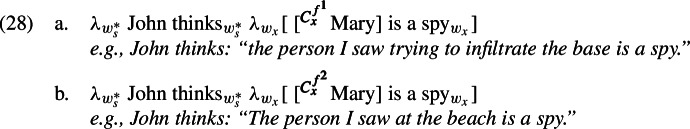


The next section provides more content and definitions necessary for how counterpart operators are formally interpreted (but readers not interested in the details can skip to Sect. 4.3). Before that, we give in (29) the LF for a sentence with a free pronoun instead of a proper name in the embedded clause. The interpretation procedure is just like it is for (27), except that the value of the embedded subject *she*_*i*_ is supplied, as usual, by an assignment function. A Counterpart function applies to it to turn it into the suitable individuals that reside in the belief worlds of John.


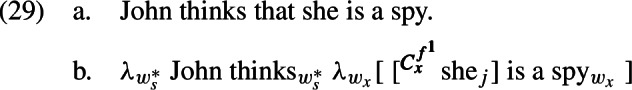


#### Counterparts: formal details

Let us first define acquaintance functions. These, adopting the spirit of Sauerland’s (2018) suggestion, are functions that relate an individual to another by some suitable description from a first-person perspective.


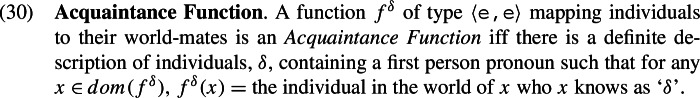


Take for instance the first-person-pronoun-containing description *δ* = ‘my math teacher’. Many individuals are acquainted with many other (world-mate) individuals through this description. When John utters it, it refers to John’s math teacher. The acquaintance function that corresponds to it, defined in (31), maps any individual *x* for which the description is defined (i.e., any *x* who knows someone as their math teacher), to the individual who fits this description for *x*.


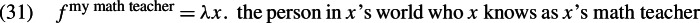


The special case of belief-based acquaintance functions is defined in (32): they guarantee a value for any *self*-counterpart of the individuals in their domain.


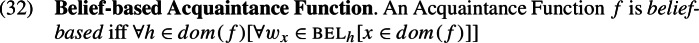
Belief-based acquaintance functions are those that are needed for attitude contexts. Informally, an acquaintance function *f* is belief-based if it preserves the underlying acquaintance relationship through which perspective-holders are acquainted with acquaintants, for all of their *self*-counterparts. Where *x* is a *self*-counterpart of some attitude holder *h*, *f*(*x*) is a world-mate of *x* and is the individual that *x* is acquainted with via the same description that specifies how *h* is acquainted with *f*(*h*).

Let us suppose further that *self*-counterparts of individuals have a unique actual Source in the following sense: every possible individual is the self-counterpart of at most one actual individual. Thus, where *h* and *j* are distinct actual individuals, there is no world-individual pair <*w*,*x*> that is in both bel_*h*_ and bel_*j*_ (see definition of bel in (26)).^11^ With this, we can explicate the semantics of Counterpart operators in (33).


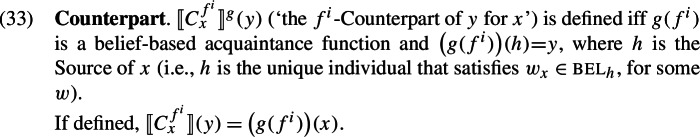


$[\!\![C^{f^{i}}_{{x}}]\!\!]^{g}(y)$ is the individual in *x*’s world who *x* is mapped to (‘acquainted with’) via the same acquaintance function that maps *x*’s Source (the attitude holder) to *y*. Note that for $[\!\![C^{f^{i}}_{{x}}]\!\!]^{g}(y)$ to be defined, *y* needs to live in the world of the Source of *x*; in the LFs in (28), for instance, *y* is the actual Mary and she lives in the world of the actual attitude holder John, who is the Source of his self-counterpart *x*. The interpretation of (34) is in (35).


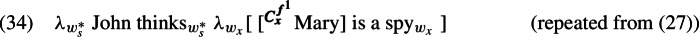



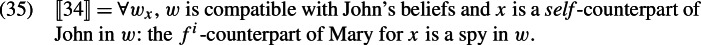


Counterparts are *f*-dependent (description-dependent), so one cannot generally talk about “the counterpart” of *y* in *w*, as there could be more than one. The same individual *y* can have different counterparts in one and the same doxastically accessible world, so $C^{f^{1}}_{{x}}(y) \not \equiv C^{f^{2}}_{{x}}(y)$ when $f^{1}$ and $f^{2}$ are two different belief-based acquaintance functions. This solves the *de re* puzzles described at the end of the previous section and allows us to capture the fact that the truth of attitude ascriptions is sometimes sensitive to the way individuals become known to attitude holders.

### The meaning of LogP and a derivation of basic sentences

The previous section provided us with tools that are rich enough to properly handle reference in attitude contexts. Now we can go back to our innovation regarding LogPs. Consider again the example in (36).


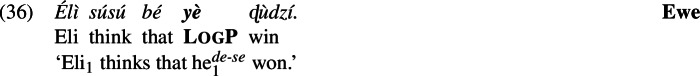


As previewed, we propose that the logophoric pronoun (in Ewe, Yoruba and Igbo) underlyingly consists of two semantically active syntactic pieces: roughly, ignoring counterpart functions for a moment, . *pro*_*i*_ is a simple individual variable with no binding requirement: it may be free or bound, and if free, its value is supplied by an assignment function. log_*x*_ is an indexed presuppositional feature, whose index must be bound by a centered-world abstractor at the edge of a clause, and is thus equated with (a counterpart of) the attitude holder.^12^


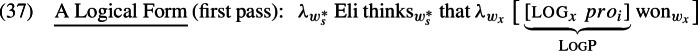


The meaning of log_*x*_ is given in (38). log_*x*_ functions like other pronominal features in being a pure-presupposition trigger (a partial identity function), acting as a filter on the possible values of its argument. Specifically, it imposes the condition that its argument’s value is identified with the index on log.^13^


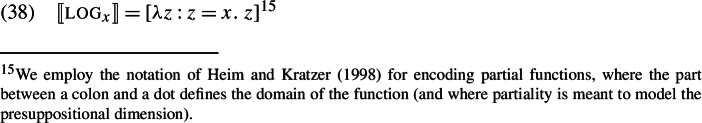


Remember that given our ontology, if *pro*_*i*_’s value is an actual-world individual—a possibility that we need to assume for the purpose of deriving strict readings—then its occurrence in an attitude context in (37) means that it must be “turned into” a counterpart of that individual that lives in the embedded world. Officially, then, we replace (37) with (39), which, just like we had in (29), appends a counterpart function to *pro*_*i*_ (but differently from (29), also has log_*x*_). Consequently, the interpretation of (29) is given in (40).


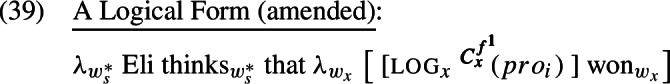



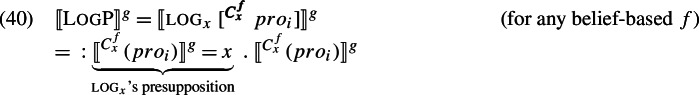


log_*x*_ thus forces the relevant counterpart of *pro*_*i*_ to be identical to the self-counterpart of the attitude holder. This is as desired, as it captures the *de se* reading of LogP: it ensures that the value of the whole LogP is the *self*-counterpart of the attitude holder (no matter how the counterpart of *pro*_*i*_ is fixed, i.e., no matter the specific value of *f*). Here’s a complete semantic derivation of (36), for an arbitrary *f*:^14^


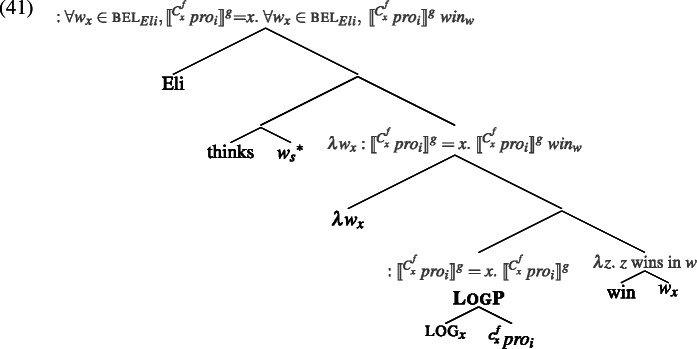



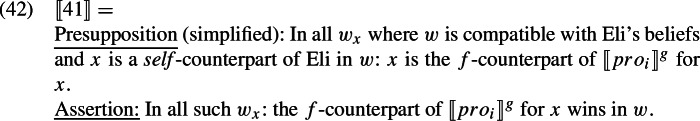


What can $[\!\![\mathit{pro}_{i} ]\!\!]^{g}$ be? Apart from contextual recoverability, free pronouns are not normally referentially constrained. But here, $pro_{i}$’s value cannot be just anyone contextually salient; if it is an actual-world individual, it must be the (actual) attitude holder—Eli. This is because only in that case will the presupposition in (42) generated by log_*x*_ be satisfied. To wit, this presupposition equates *x*, a *self*-counterpart of Eli, with a counterpart of $[\!\![\mathit{pro}_{i} ]\!\!]^{g}$. By definition of Counterpart in (33), possible individuals have only one Source, which means that the Source of *x* must be the Source of $[\!\![\mathit{pro}_{i} ]\!\!]^{g}$ as well. From these two, and from the fact that Eli is the Source of *x*, it follows that $[\!\![\mathit{pro}_{i} ]\!\!]^{g}=$ Eli. Therefore we can safely replace (42) with (43):


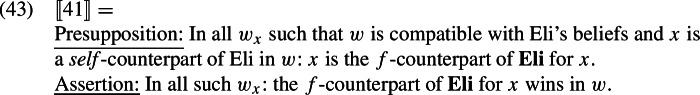


Now what can the value of the acquaintance function-variable *f* be? It too, in principle, merely needs to be recoverable from context (by some salient-enough description); but here too, *f* is semantically restricted: only those values are possible that result in a presuppositional statement that can be safely accommodated. In our case, because $[\!\![\text{$pro_{i}$} ]\!\!]^{g}$ must be Eli, *f* must in turn be an acquaintance function that maps Eli to himself both in the actual world and in his alternative worlds. The option in (44a), which is just the identity function (underlined by the *se* description, “me”), will always be possible here; also the one in (44b) is ok in natural contexts in which Eli knows himself as the person called ‘Eli’. Some possibilities are out in most natural contexts since they would incur a presupposition failure, e.g., (44c) (if Eli ≠ Ann); and others are heavily context-dependent, for instance (44c), which would satisfy the presupposition only in contexts where Eli wears a red costume and identifies himself as such.^15^ This nicety in the options for *f* will become relevant soon.


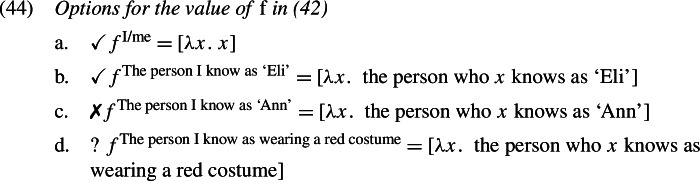


### Strict readings by ignoring log from alternatives

We have just shown how to derive obligatory *de se* readings by splitting up LogPs and by assuming that the log feature introduces a presupposition (without any formal binding dependency between its $pro_{i}$ part and the matrix subject). By this, we merely replicated a basic result already obtained by Simplex Binding, just in a more complicated way. But the complication allows us to derive strict readings, to which we now turn. Consider again an example that brings strict-sloppy ambiguity to light. We repeat example (11) in an abbreviated form in (45), with which we will exemplify our analysis.


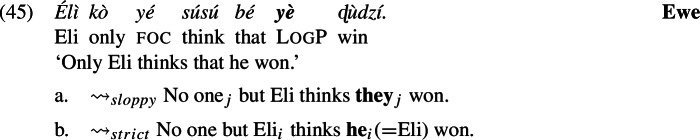


We continue to analyze these constructions as involving computation of focus alternatives triggered by a focus feature on the subject, as represented in (46a). For concreteness we take alternatives to be syntactic objects, LFs (Fox and Katzir 2011). *only* says that the prejacent (its sister) is true and all the alternatives are false.^16^ Now if log contributes its meaning across the focus alternatives, as represented in (46b), we are still short of deriving the intended strict reading.


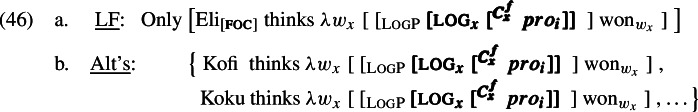


In particular, (46) fails at the level of focus alternatives. To appreciate why, notice first that since $pro_{i}$ refers to Eli, which as we have shown above is forced on us by the presence of log at the prejacent, then $pro_{i}$’s reference to Eli will remain constant across the alternatives. But then, by the same reasoning, log’s presence in the alternatives results in the false information that the relevant alternatives to Eli (Kofi, Koku) are in actuality equated with Eli, which cannot be.^17^

However, we assume as discussed in Sect. 4.1 that log—being a pronominal semantic feature—is subject to the hypothesis in (23) and thus can be ignored when computing focus alternatives, like fake *ϕ*-features on bound pronouns. We implement the idea by letting log be deleted from the tier of alternatives (though not from the prejacent). The relevant derivation is in (47).


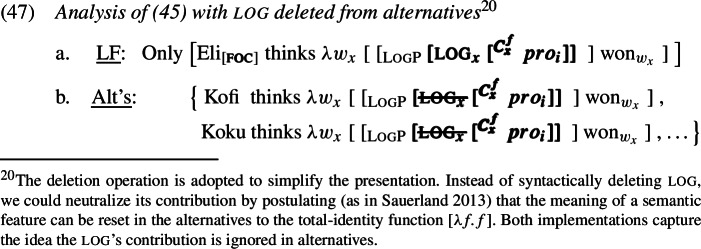
As above, the referent of $pro_{i}$ remains Eli across the alternatives. But since log is active only in the prejacent now, the offending presupposition is absent in the alternatives. The interpretation of this configuration is given in (48), and *only* negates the alternatives.


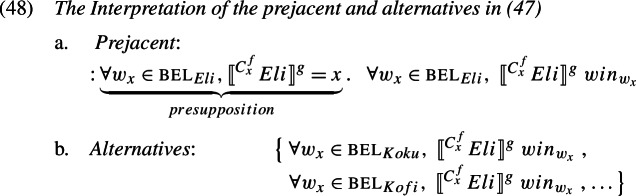


The specific interpretation of this depends, of course, on the specific acquaintance function chosen for *f*. All that is required is for *f* to represent an acquaintance function that links Eli and all of his alternatives (Koku, Kofi, …) to Eli, and that furthermore they all mentally associate with Eli. If, for example, it is common ground that everyone knows Eli by the name ‘Eli’, then plugging in for *f* the value in (44b), will result in a suitable strict reading.

This proposal makes the correct prediction that the strict reading can be achieved via many different ways of specifying *f*. Koku and Kofi do not have to be acquainted with the referent of LogP through the description ‘the person called Eli’; they only need to know him through some shared description, if one can be accommodated. That the prediction is borne out is exemplified in (49). In this scenario, Koku and Kofi are acquainted with a certain man in a red costume, but do not know it is Eli. We call it a Strict-Unknown scenario.


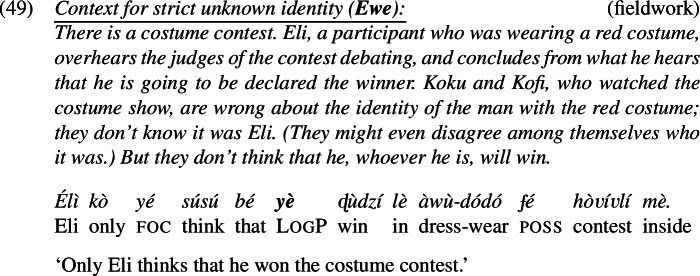
Our Ewe consultants judged the sentence felicitous and true in the scenario. The sentence entails that Koku and Kofi don’t think that the man in the red costume won—despite their lack of awareness that it is Eli.

The facts hold in Igbo and Yoruba as well. In Strict-Unknown scenarios, LogP *yá* in Igbo and LogP *òun* in Yoruba are felicitous; see (50) and (51).


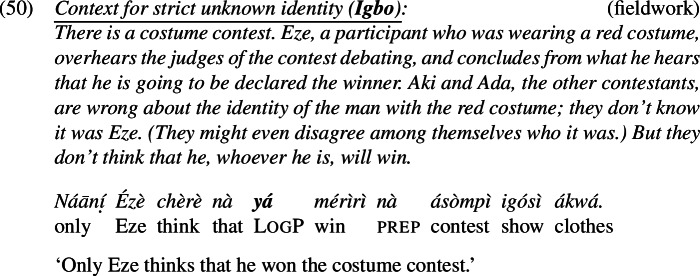



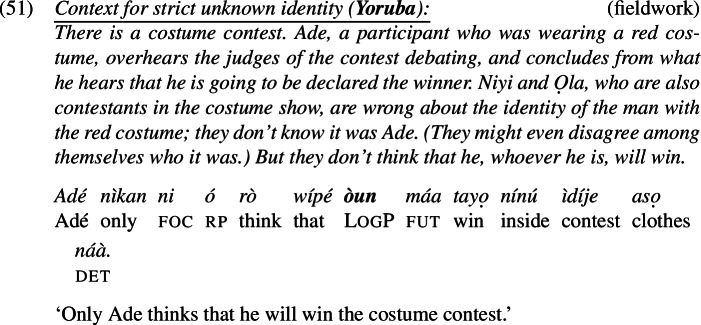


The reason LogPs are predicted to be licensed in strict-unknown cases is that the value for *f* in our LF configuration in (47) can be resolved to the description in (44c). This description is salient in the context, and it refers to Eli in Eli’s mind but not to who Koku and Kofi associate with the description ‘Eli’.^18^

As one of our consultants emphasized, if Eli does not know that he is the man in the red costume, the target sentence in (49) is not felicitous in such strict-unknown scenarios. Thus, the attitude holder in the prejacent (*Eli*) still has to be familiar with himself as the man in the red costume. This is expected for the same reason that *de re* readings of LogPs are out in basic sentences (cf. Sect. 2.2): log’s presupposition, which in the prejacent cannot be ignored, imposes the *de se* condition on the use of LogP. This concludes our basic proposal of the strict reading of LogPs.

### Sloppy readings

As for the sloppy reading of LogPs, we can derive it by binding the variable part (*pro*_*i*_) of LogP directly to the matrix subject, as in the representation in (52), and setting the value of *f* to be the identity function [*λx*. *x*] (underlined by the *se* description “me”; see (44a)). Since the binding dependency persists across the alternatives, the value of *pro*_*i*_ co-varies in alternatives with the respective attitude holder. The sloppy reading thereby obtains (in fact, whether or not log gets suspended in alternatives).


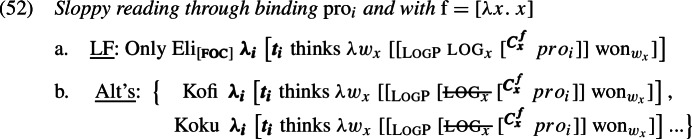


### Strict ellipsis

We have applied our analysis to sentences with focus and *only*, though earlier we saw that strict readings for LogPs show up in ellipsis constructions as well. Recall, e.g., (53), repeated from (16).


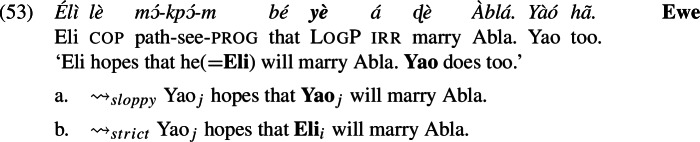


Ellipsis licensing requires an antecedent with a parallel meaning. We follow Tancredi (1992), Rooth (1992a), Takahashi and Fox (2005), Merchant (2019) and many others in linking the Parallelism requirement to the theory of focus:


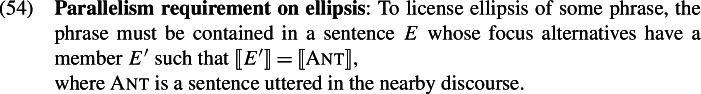


The special status of log as a pronominal feature in our theory helps with the strict reading in ellipsis, too. We will adopt a version of Sauerland’s (2013) proposal on alternatives to work for ellipsis. We stipulate that presuppositions coming from pronominal features in an antecedent Ant may be ignored for the purpose of computing the identity statement for ellipsis. Thus, we replace (54) with (55):


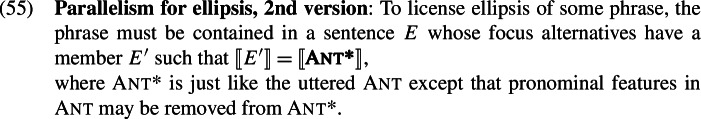


Parallelism is satisfied if what is elided is not a LogP but the more deficient OrdP: a pronoun with the same referential core $pro_{i}$ but without the log feature (below we abstract away from counterpart operators, to simplify the representations and because they are irrelevant to the point).


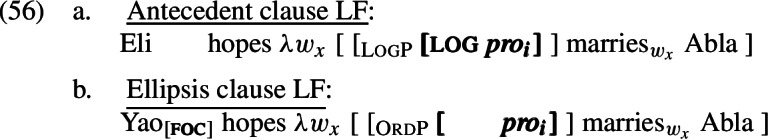
The two clauses satisfy our version of the Parallelism requirement, as follows. One of the focus alternatives of the Ellipsis clause in (56b) is the one where *Yao* is replaced by *Eli*, in (57).


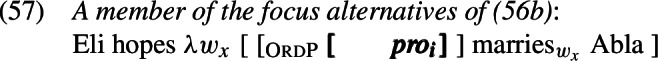
Indeed, (57) is derived from (56a) by removing the feature log, and therefore it is an appropriate Ant*. Ellipsis is consequently licensed by condition (55).

Like before, because we have a LogP in Ant, the value of $pro_{i}$ is restricted by the log feature to be Eli. The ellipsis clause must then contain an occurrence of the same variable in that position (the underlying acquaintance function *f* could be, for example, “the person called ‘Eli’’’, assuming both Eli and Yao associate this person with Eli).

## Further consequences of the account

This section explores consequences of our theory regarding two other properties of logophors: their ability to take long-distance antecedents in multiple attitude sentences (Sect. 5.1), and their complementary distribution with the first person pronoun (Sect. 5.2). The account we offer in Sect. 5.2 captures an observation which, as far as we know, has not received adequate attention (let alone an explanation) in the formal literature, namely why LogPs cannot be anteceded by first-person attitude holders (Hagège 1974; Hyman and Comrie 1981).

### Long-distance antecedents

In this section, we analyze the ability of logophors to take long-distance antecedents. As we show in (58)-(60), LogPs embedded under more than one attitude predicate can co-refer with either the local attitude holder or the more distant attitude holder. Overall, the co-reference patterns are stable across attitude predicates and languages.^19^ Our observations are in line with what has been reported for Ewe and Yoruba in previous literature (Clements 1975; Manfredi 1987; Anand 2006; Pearson 2015). We further contribute multiple embedding data from Igbo, which patterns with Yoruba and Ewe.


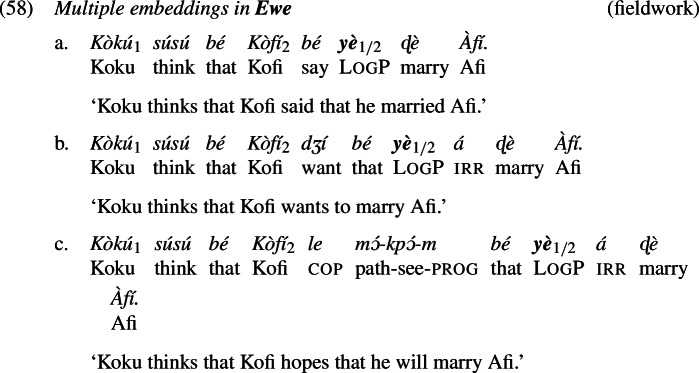



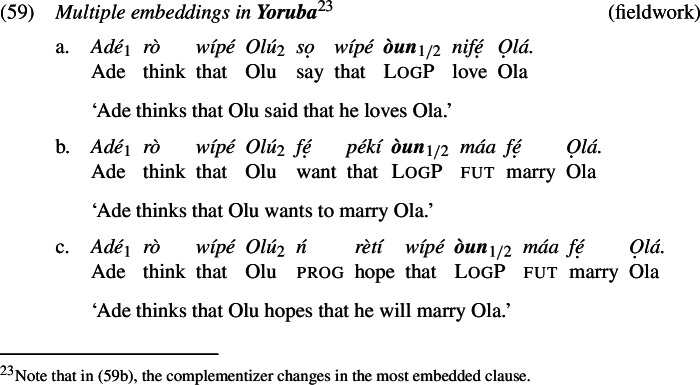



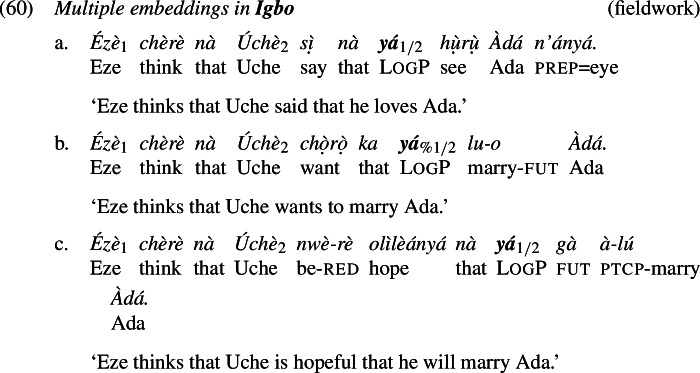


We have the tools to account for the option of LogPs to take long-distance antecedents: both attitude predicates introduce complement clauses and therefore abstraction over centered worlds, so in principle either can bind LogP. More specifically, either can bind the index on log. Taking sentence (58b) as representative, and leaving out counterpart operators for a moment, we assume that it can correspond either to representation (61a) or to representation (61b), where the difference is underlined.


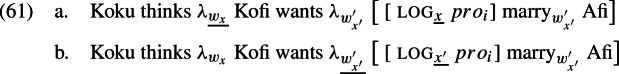
But not quite; once again, our background ontology and semantics require the representation to be decorated with counterpart operators. We will disregard any counterpart operators that in both LFs need to be inserted anyway on the individual-denoting phrases *Kofi* and *Afi*, and restrict attention to those that need to be inserted inside LogP. For (61b), which is intended to capture the local-binding option, a corresponding counterpart operator with the same index as on log is added, so that $pro_{i}$ ends up referring to a counterpart of the embedded subject (Kofi); this is shown in (62b). For (61a), which is intended to capture the long-distance reading, a suitable operator is analogously added on $pro_{i}$, and in addition the whole LogP is appended with a further counterpart operator bound by the embedded subject, so that LogP as a whole can denote a counterpart (of a counterpart of the matrix subject Koku) that resides in the embedded-bound worlds $w'$. This is shown in (62a).^20^


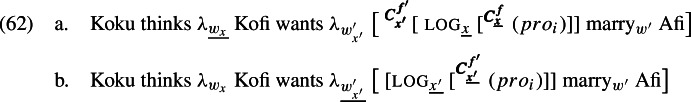


We point out a certain welcome consequence of this complex representation of the long-distance configuration in (62a). Pearson (2015, p. 111, exx. (91)-(92)) shows that while long-distance-bound LogP is read *de se* with respect to its antecedent, the matrix attitude holder, it may be read in various *de re* ways with respect to the attitude of the intervening attitude holders (she demonstrates that using embedded double-vision scenarios; we leave out her example for space reasons). Let’s see how this fact is precisely captured by the layered arrangement of counterpart operators in (62a).

The meaning the current system assigns to (62a) is approximated in (63). Due to log’s presuppositional contribution, LogP ends up referring to a *self*-counterpart of the matrix subject (Koku), guaranteeing the *de se* dependency with it.^21^


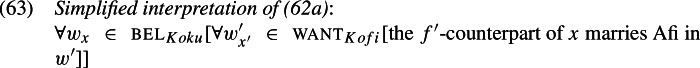


The *de se* dependency, however, doesn’t constrain how Kofi needs to be acquainted with Koku, through $f'$. Different ways to specify $f'$ represent different ways for how Kofi is acquainted with Koku (according to Koku). If, for example, (Koku thinks that) Kofi is ordinarily acquainted with him through the description ‘the person that I know as “Koku’’’, and it is this description that underlies $f'$, then the result is the mundane construal in (64).


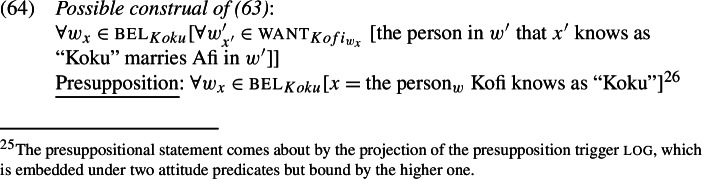


But imagine a context where Kofi knows Koku only through the description ‘my math teacher’—or at least, that this is what Koku believes to be the case. That is, Koku thinks “this guy Kofi doesn’t know that my name is ‘Koku’, he only knows me as his math teacher.” This scenario specifies a different counterpart function than before, and the resulting construal is now:


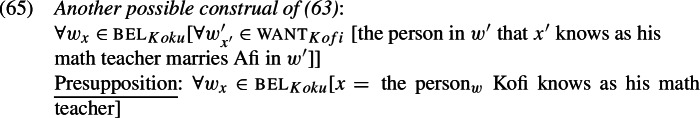


The upshot is that in long-distance configurations, we predict that LogP is restricted to refer *de se* to its antecedent (the matrix attitude holder), but can be read in various unconstrained ways with respect to the beliefs of intervening attitude holders—as borne out by the discussion in Pearson (2015, p. 111).^23^

### Competition between LogP and the first person pronoun

We end with a proposal about the logophoric pronoun’s restriction to occurrences in complements of attitude predicates. LogPs cannot typically appear unembedded:


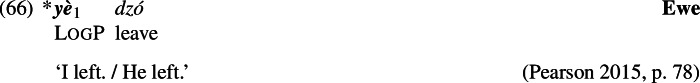


But on some assumptions detailed below, our approach currently predicts that (66) is grammatical and that the LogP refers to the speaker, i.e., that (66) means “I left”. LogPs in the languages under discussion, however, cannot refer to the actual speaker; instead, there is a dedicated first person pronoun:


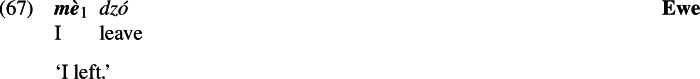


We offer a new perspective on this restriction, which crucially relies on our novel presuppositional semantics for LogPs, together with the competition principle *Maximize Presupposition!* (Heim 1991, et seq.): in a nutshell, LogP competes and loses to a first person pronoun whenever the latter can be used without change of meaning.

Previous proposals encode the restriction exemplified in (66), with the stipulation that LogPs must be bound at the edge of complement clauses, by attitude verbs only (von Stechow 2004; Heim 2005; Pearson 2015), but we show below that our alternative account allows us to simplify the grammar of LogPs somewhat and replace that stipulation with the weaker demand that LogPs must be bound at the edge of some clause—not necessarily complement clauses.

We will then show that the *Maximize Presupposition!*-based proposal has an empirical advantage over the abovementioned accounts in that it can account for a hitherto unexplained generalization: even in attitude environments, LogPs are out if anteceded by first-person attitude holders (Hagège 1974; Hyman and Comrie 1981).


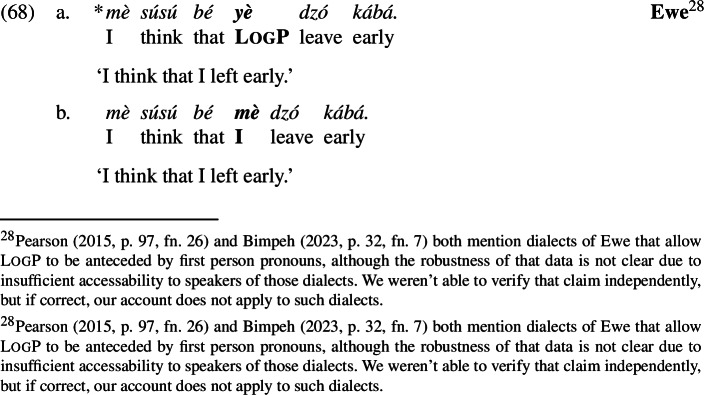


#### What LogPs denote in matrix environments

Recall from Sect. 4.3 the denotation we assign to a LogP, repeated in (69).^25^


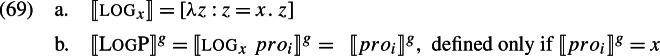


We assumed there (see fn. 13) that LogPs—more accurately, the index on log—must be bound by an abstractor over centered worlds from the edge of a clause.^26^ If so, nothing prohibits a matrix occurrence of a LogP where it is bound by the top-most centered-world abstractor introduced at the matrix level. The result would be:


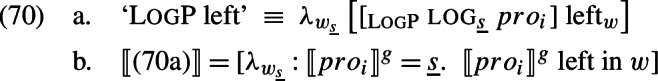


The world coordinate of a matrix centered world corresponds, as standard, to a world compatible with the actual context of utterance, and it is natural to take the individual coordinate to be the speaker of the context. This is meant to capture the intuition that the “logophoric center” of a matrix sentence is the speaker of the sentence. We technically implement this with the discourse rule in (71) relating the proposition expressed by a sentence to contexts in which the sentence can be appropriately uttered by speakers (the notions of Common Ground and Context Set in (71a) are adapted versions of their familiar predecessors from the works of Stalnaker 1978, et seq.)


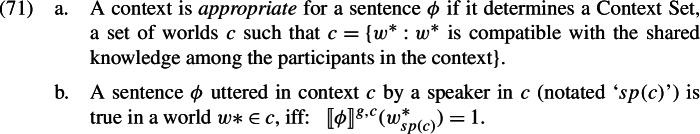


Given all this, (70a) is predicted to be grammatical and appropriately used to express the proposition that the speaker left, wrongly so (cf. (66)).

#### Competition via *Maximize Presupposition!*

In principle, we could avoid the problem with a syntactic stipulation to the effect that LogPs are licensed only when bound by attitude predicates, like previous approches assumed (see Sect. 3.1). But, as previewed, we want to offer an alternative that doesn’t require this restriction, and to derive the infelicity of (66) as the result of competition with (67).

To do so, we need a concrete analysis of first person pronouns. We take the first person pronoun to contain a variable $pro_{i}$ and a semantic feature 1st whose job is to restrict the variable’s value to be the speaker, as represented in (72). This much is fairly standard (Heim 2008, Charnavel 2019, a.o.), but we also assume that 1st, like log, comes with an index that must be bound by a logophoric abstractor. The index’s value in this case is identified with $pro_{i}$ and, unlike log, also with the actual speaker. The lexical entry of 1st, a purely presuppositional function, is thus in (73a).^27^ (‘*c*’ stands for the Context Set as was defined in (71a).)


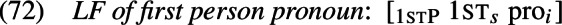



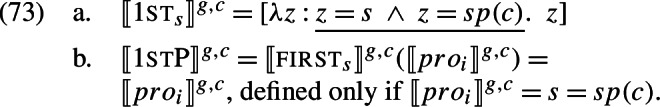


Below is the LF and meaning of (67) ‘I left’.


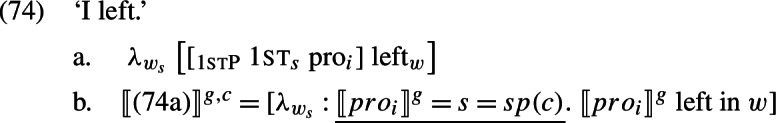


The resulting partial proposition in (74b) has a stronger semantic presupposition—a strictly smaller domain—than the LogP version of that sentence in (70b). While (70b) is in principle defined for any individual *s*($=pro_{i}$), (74b) is defined only for the actual speaker(= $pro_{i}$). But when defined, both (70b) and (74b) have the exact same assertive content and therefore convey the same information: the speaker left. A situation where two alternative propositions have the same assertive content but one has a stronger presupposition is generally taken to feed the competition principle *Maximize Presupposition!* (Heim 1991; Percus 2006; Sauerland 2008; Schlenker 2012), with the consequence that the presuppositionally weaker alternative is blocked.


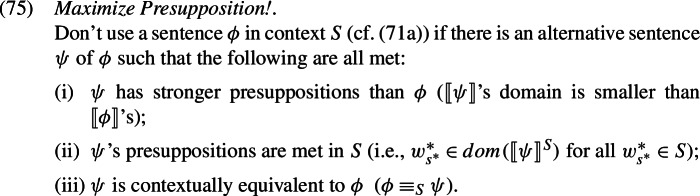


We assume that 1stP is formally an alternative to LogP (as it is syntactically no more complex than it; Katzir 2007) and therefore *Maximize Presupposition!* dictates that a sentence with LogP cannot be used to refer to the speaker.^28^ This explains the restriction in matrix environments.

#### 1st-log competition in embedded positions

First person pronouns are, of course, licensed not only in matrix positions but also in embedded ones. For example, *John thinks that I left*, a suitable analysis within our formalism is given in (76a), with its assigned interpretation in (76b). The presupposition of 1st, once again, guarantees that the reference is to the (relevant counterpart of) the actual speaker. Note that the semantics of 1sc forces the index on 1sc to be bound by the matrix abstractor over centered worlds, and that a counterpart operator must consequently be added.


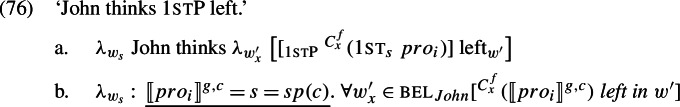


We can now proceed to suggest an explanation for the restriction on first-person antecedents, repeated from (68) (the facts hold in Yoruba and Igbo as well):


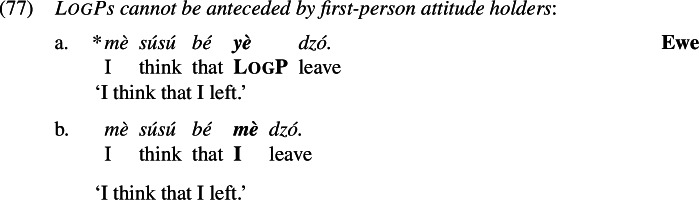


Why is (77a) not acceptable? The answer, we propose, is that (77b) blocks (77a) by *Maximize Presupposition!*, just like (74b) blocked (70b) in matrix environments. Here is the LF-analysis of the two sentences:


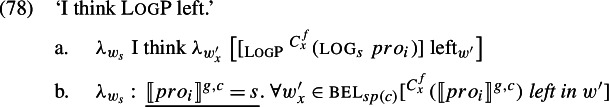



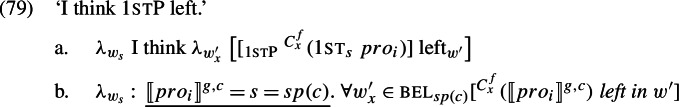
The underlined parts reveal again that (79) has a strictly stronger presupposition than (78). But they have the exact same assertive content. Therefore the latter is blocked by *Maximize Presupposition!*.^29^

While this competition-based account explains (77) as a direct extension of the account of matrix environments, it is difficult to see how previous accounts of the dependency between a LogP and its antecedent can capture it without further stipulations. If LogPs are merely syntactically required to be bound by an attitude verb (as is assumed by Simplex Binding, Sect. 3.1), then additional mechanisms are needed to ensure that a first-person subject is not a possible antecedent.^30^

## Conclusion

This paper provided evidence that logophoric pronouns (LogPs) in Ewe, Yoruba and Igbo support both strict and sloppy readings in ellipsis configurations and in sentences with *only* (following observations in Culy 1994; Bimpeh and Sode 2021), and offered a formal analysis that could capture this behavior. The account supplants existing accounts of LogPs with the idea that LogPs are pronouns that contain a semantic feature log in charge of encoding the *de se* reference to the attitude holder (following Bimpeh et al. 2024), but whose contribution can be ignored at the level of focus alternatives, like other pronominal features. We also showed how long-distance dependencies of LogPs are handled, and provided a novel solution for the restriction on the occurrence of LogP in matrix environments and with first-person antecedents.

## Supplementary Information

Below is the link to the electronic supplementary material.

(PDF 173 kB)

## Footnotes

1 Glossing abbreviations follow the Leipzig glossing rules, with the addition of LogP = logophoric pronoun, OrdP = ordinary pronoun, rp = resumptive pronoun, prep = preposition, and red = reduplication.

2 Given that we tested the distribution of LogP and OrdP across several predicates, the actual number of test items per data point was never more than 2. As for *de se*/*de re* readings (Sect. 2.2), strict/sloppy identity (Sect. 3) and multiple embeddings (Sect. 5.1), we tested the distribution of logophors and pronouns embedded under the verbs ‘say’, ‘think’ and ‘hope’ consistently with all our consultants. The verbs for ‘want’ were tested with all speakers for *de se* readings. We also tested with the verbs for ‘promise’ in all three languages (see Supplementary Material). For strict unknown identity, we only elicited judgments with the verbs for ‘think’.

3 Although tense in Ewe (as well as in most Kwa languages) is not overtly marked, Ewe displays an irrealis marker *a* that expresses the possibility of an event occurring in the future (Essegbey 2008). The irrealis marker *a* can be optionally added in (3a) and (3b) to express such a meaning. In (3c) and (3c), however, the marker occurs obligatorily. The environments where the irrealis marker occurs obligatorily roughly match the ones known in English as complements of control verbs; see also the Supplementary Material and the discussion in Grano and Lotven (2019) for Gengbe, an Ewe dialect spoken in Togo.

4 In contrast to Ewe, the forms for LogP in Yoruba and Igbo are taken from the general paradigm of strong pronouns, and may therefore also occur in unembedded environments. The strong form *òun* in Yoruba is used in emphatic environments, while the strong form *yá* in Igbo is used additionally in object position (Pulleybank 1986; Manfredi 1987; Amaechi 2020).

5 Nor do we intend to cover in any depth the connection between LogPs in West African and other phenomena sometimes discussed under the general rubric of “logophoricity”: perspectival anaphora (Sundaresan 2018), exempt anaphora (Charnavel 2020), and perhaps Indexical Shift (Deal 2020). But see Sect. 4 in the Supplementary Material for a comparison to the phenomenon of exempt anaphors.

6 Given that Pearson (2015) and other works reach the opposite conclusion to ours for Ewe (using different methodology), a reviewer suggests that one cannot rule out the possibility that there is a dialect split or cross-speaker variation in Ewe. On this point we side with Pearson (2022), who contends that it is unclear what sort of evidence in the input could trigger the acquisition of different grammars by different speakers on the *de re* property of LogPs. Moreover, we hope that our methods for eliminating possible confounds will prove useful in clarifying the empirical picture (for Ewe). But if it turns out that a genuine variation is what underlies the disagreement in the literature, then our analysis from hereon should be taken to apply only to those speakers (like ours) who only accept *de se* readings of LogPs; if there are speakers whose grammar truly allows *de re* construals of LogP, our account would have to be enriched with further mechanisms to accommodate them.

7 There are differences in implementation between Pearson’s proposal and the other works cited above, but those differences are immaterial for us insofar as we are merely interested in the Simplex Binding assumption that these accounts are all committed to.

8 Our proposal is most directly inspired by Sauerland’s (2013) approach to the phenomenon of strict reading of reflexive pronouns (see Sag 1976; Fiengo and May 1994; Hestvik 1995; McKillen 2016, a.o.). Reflexive anaphors, e.g., English *self*-pronouns in local configurations, permit strict readings in ellipsis and focus environments: Sauerland derives the strict reading from the idea that reflexive anaphors contain a self morpheme with presuppositional semantics that can be ignored in focus alternatives.

9 The core proposal in this paper for the syntax and semantics of LogPs is, we believe, largely orthogonal to the general problem of reference in attitude contexts. It should thus be kept in mind that some of the particular theoretical choices we make in Sect. 4.2 are made largely for concreteness.

10 We will discuss the contribution of the matrix abstractor $\lambda{w^{*}_{s}}$ (roughly over actual world-speaker pairs), and its relevance to the grammar of LogPs, in Sect. 5.2. Until then, it will not play much role.

11 This assumption is defensible insofar as we hold that no two individuals have identical experiences and completely compatible beliefs about the world. It thus ignores metaphysical edge cases such as the two-gods scenario discussed in Lewis (1979, p. 520).

12 We are deliberately saying that the index on log_*x*_ must be bound at the edge of a **clause**, not necessarily at the edge of a **complement** clause as is usually assumed in accounts of logophoric pronouns (von Stechow 2004; Pearson 2015, a.o.). The reason for this more relaxed requirement will be discussed in Sect. 5.2, where we show that it can help explain a further distributional property of LogPs.

13 A reviewer asks in what sense the log feature can be counted as a pronominal feature. Semantically, as we lay out in the next section, our denotation for log adds a presuppositional restriction on the value of a variable—in that sense it matches what has been proposed about the semantics of *ϕ*-features. And syntactically, there is evidence that logophors trigger dedicated logophoric agreement on the verb in languages like Ibibio, shown for the logophor  in (ia). Hence, the log feature behaves morpho-syntactically like other *ϕ*-features in this respect. While this property cannot be investigated in the languages we focus on in this paper since Ewe, Yoruba and Igbo do not display subject verb agreement, we note that in the languages with logophors that do display subject verb agreement, dedicated inflectional morphology is attested, as one would expect from a pronominal feature.

14 Given that we now represent presuppositions, we replace the entry for *think* we had in (25b) with the one in (i) that encodes how presuppositions are projected, adopted after Karttunen (1974) and Heim (1992). This entry is used in the calculation in (41). Similar lexical entries need to be written for predicates of speech, desire, hopes etc. All that matters for our purposes is that for each such predicate the domain of quantification is a set of centered worlds, and that the presupposition of log projects to bel.

15 Acquaintance functions that don’t map Eli to himself in the actual world (i.e., acquaintance functions that are not *reliable* in the sense of Pearson 2015) would not result in a satisfied presupposition in (41), even if Eli mistakenly associates himself with that description. Imagine for instance that Eli believes he’s the person wearing the red costume, but is mistaken about it (he actually wears a different costume). In that context, (44c) cannot be the value for *f* given LF (41).

16 We evidently assume in (46a) that *only* scopes at LF over the whole rest of the clause. This is done for simplicity’s sake. We could instead adopt the bipartite structure *[[only DP*_*Foc*_*] VP]* (Wagner 2006, a.o.), where *only* forms a constituent with its focused associate (the subject). Our main proposal isn’t affected by this choice, as long as the subject DP formally triggers focus alternatives.

17 (46b) also results in the information that the alternatives Kofi, Koku identify themselves in their mind with Eli (this part by itself is not contradictory).

18 What if a context furnishes no suitable definite description that could be the value of *f* in this derivation? Our predictions might change. Imagine we cook up a context where it is impossible to find any (salient) description that links Eli and all his relevant alternatives to Eli and is vivid enough in their minds (i.e., across the doxastically accessible worlds) as such. In such a context, we predict that sentence (45) would not support the strict reading (we thank Amir Anvari, p.c., for raising a similar point to us). We think, however, that finding convincing cases of such contexts is not trivial, as there is arguably always *some* shared description that can be accommodated whose reference in the minds of the relevant individuals would be the matrix subject. In the absence of independently justifiable constraints on what concepts can be accommodated in a context, it is not entirely clear to us how to create the relevant scenarios. We leave this as an open question for now.

19 There was one exception. For the multiple embedding including ‘want’ in Igbo (60b), one of our consultants did not accept the co-reference reading with the long distance antecedent.

20 If we didn’t have the outermost counterpart in (61a), we would have the illegitimate configuration in which the subject of the most embedded predication resides in the matrix-bound worlds (*w*). Remember that given the ontology, an individual-denoting phrase needs to denote an individual that resides in the worlds where the local predication takes place, in this case $w'$.

21 As mentioned, a more complete LF requires counterparts on the other individual-denoting phrases here. In particular different options for the resolution of Kofi’s counterpart in the belief worlds of Koku (the matrix attitude holder) would yield different results per how Koku is acquainted with Kofi. This doesn’t affect the point we are makin here in any substantial way. We should note here that multiple embeddings on our account requires a certain modification of the definitions of “Counterpart” and “Source” from (33), to allow not just for actual individuals but also for possible individuals to have counterparts and to be sources of others. The extension is straightforward but cannot be discussed for space reasons.

23 Although, Pearson (2015, p. 112) remarks in passing that LogPs in such sentences can, but don’t have to, be construed *de se* when bound long-distantly. She does not provide evidence, but that’s in line with her general claim about LogPs; see Sect. 2.2.2. For us, *de se* with respect to the matrix holder is a necessity.

25 Counterpart operators are not needed in matrix environments, so they are left out here.

26 If we didn’t place any binding restrictions on log, we would wildly overgenerate readings, because then it wouldn’t be clear what rules out a LogP with a free-indexed log from referring to some contextually salient individual.

27 This analysis of course is not applicable to languages with Indexical Shifting, as the first person pronoun can also pick out attitude holders in those languages (see Deal 2020). In fact, if our analysis is to be extended to indexical shifting languages, then the first person pronoun in those languages can be analyzed the way we analyze LogPs in West African (and moreover, indexical shifting languages would have to be assumed to lack a pronoun with the syntax and semantics in (72)-(73)).

28 *Maximize Presupposition!* has been invoked to explain certain inferences arising from the use of the presuppositionally weaker alternative (“anti-presuppositions”; Percus 2006, a.o.). In our case, however, no anti-presupposition can be detected from a use of unembedded LogP because the relevant presupposition of the stronger alternative 1stP is always met; more precisely, a hypothetical context in which that presupposition isn’t met would be one where the sentence as parsed in (74a) could not be uttered by the speaker in the context to begin with, given the definition of appropriate context in (71).

29 In (78a) the index on log is bound by the matrix abstractor ($\lambda _{w_{s}}$). Things change if we choose the index *x* bound by the embedded abstractor ($\lambda _{w'_{x}}$). That parse, in (i), generates the presupposition underlined in (ib). This amounts to the presupposition that across the speaker’s belief worlds, her *self*-counterpart is also the counterpart of *pro*. We are not sure at the moment what blocks this representation.

30 The logic of the account of (77) leads us to expect that LogPs are fine with second-person antecedents. While this is correct for Ewe (Clements 1975; Bimpeh 2023) and other languages (see Hyman and Comrie 1981; Stirling 1993; Nikitina 2012), it appears to not be true for all logophoric languages (see, e.g., Kiemtoré 2022). We leave this issue unresolved for now and hope to revisit it in a future occasion.
