# Supplementary material for: Decomposing logophoric pronouns: a presuppositional account of logophoric dependencies
Source: Nat Lang Semant. 2025 Oct 30;33(4):663–702. doi: 10.1007/s11050-025-09242-x (PMC12680770; doi:10.1007/s11050-025-09242-x)
Supplement: Supplementary file 1 — (PDF 173 kB) [file 11050_2025_9242_MOESM1_ESM.pdf]

Supplementary Material

(Decomposing logophoric pronouns: a presuppositional account of logophoric dependencies)

1 Language profiles

All three languages are spoken in West Africa and belong to the Niger-Congo language families. Ewe (indigenously pronounced Eʋe) is a Niger-Congo language of the Kwa group that is a member of the larger unit of closely related languages known as the Gbe languages. According to Capo (2010) and Ameka (1991), Gbe 'voice / language' refers to a dialect cluster comprising Gen, Aja, Xwla-xweda (Phla-Pherá), Fon, and Ewe. Speakers of the Gbe languages inhabit the Volta and Oti regions of Ghana, the southern part of Togo, the southern part of Benin, as well as parts of Ogun and Lagos state, Nigeria. Ewe is a cover term used to refer to a group of (sub)dialects spoken in the Volta and Oti regions of Ghana by about 2.5 million people, and in the southern part of Togo by approximately 1 million people.

Yoruba is a dialect continuum spoken in the area that covers Western Nigeria and Lagos, the Ilorin province of Northern Nigeria, parts of Benin, Sierra Leone, Cote D'Ivoire and the Gambia. The language belongs to the Benue-Congo language family and is spoken by 44.6 million speakers (Eberhard et al., 2020). According to Bamgboṣe (1966), Yoruba comprises about twenty dialects such as Ijèbú, Egbá,

Ijèsà, Oyó, Owó, Ondó, each of which differs considerably from the other phonologically, and lexically. There is a standard variety of Yoruba, based on the Oyó dialect, called Koine which is used for the purpose of education, writing, and contact between persons of different dialects.

Igbo is the principal native language of the Igbo people, an ethnic group from Southeastern Nigeria, and the second most populous indigenous language of that area. Other Igbo speaking areas include Equitorial Guinea and Cameroon. The Igbos are one of the three largest ethnic groups in Nigeria (Awde and Wambu, 1999). They are predominately located in five states namely, Anambra, Imo, Ebonyi, Abia, and Enugu. A significant number of Igbo communities can also be found in neighbouring states such as Rivers, Asaba as well as the commercial centre of the country, i.e., Lagos (Awde and Wambu, 1999). The language belongs to the Benue-Congo language family and is spoken by 2.2 million speakers (Eberhard et al., 2020). There are dozens of geographical dialects. These dialects differ in certain grammatical, lexical and phonological details (Emenanjo, 2015). The standard dialect is based mainly on Owerri, Umuahia and Onitsha dialects.

2 Obligatory *de se* readings of logophors

This section provides more details and data on the elicitation of *de re* vs. *de se* scenarios. As discussed in section 2 of the main paper, one aspect of our methodology includes the inclusion of ORDPs in our target sentences when tested in *de re* contexts (mistaken identity scenarios). Another modification on our side is the fact that both LOGPs and ORDPs were tested in *de se* scenarios. The *de se* contexts were designed to be minimally different from the *de re* contexts. With *think* and *say* specifically, we created contexts which were equally elaborate in order to test whether the complexity of the scenario influences the judgement. The complexity did not interact with the judgements of our speakers. We present the results for the *de se* contexts in (1)-(4),

along with the *de re* counterparts, repeated from the paper, in (5)-(8).[1] Interestingly, languages differ regarding the availability of OrdP in *de se* contexts. Whereas in Ewe OrdP is unacceptable and only the logophor is licensed, in Yoruba and Igbo both LogP and OrdP are acceptable. Given the data in (1)-(4), in particular the availability of OrdP in Yoruba and Igbo, we conclude that pronouns can indeed be ambiguous between *de se* and *de re* readings, and if they are, our methodology will track such ambiguity. In other words, we can exclude the possibility that logophors might be ambiguous after all, but a general bias for pronouns towards *de se* readings makes them unacceptable in *de re* contexts (cf. Pearson, 2022). If this were the case, we would not expect OrdP to be acceptable in both *de se* and *de re* contexts in Yoruba and Igbo.[2]

(1) *De se context, think:* *(Bimpeh et al., 2024)*
Donald Duck (DD) went to the grocery store to buy flour. Then, he mistakenly put sugar in his cart. DD went on and then, he saw a trail of sugar going up and down the aisles and thought that someone's bag had a hole in it and looked around for the guy. DD says: "I wonder who is losing sugar" "Certainly, the guy who is losing sugar is stupid, and it is not me because I bought flour not sugar!" Later he says "But I did not check!" "Let me see if it's me the stupid guy who is losing sugar." He checks in his bag and sees the sugar. Finally, he realised.

a. Donald Duck súsú be **yè** / **#é** dzɔ-mo-vi. **Ewe**
Donald Duck think that LogP / OrdP exist.with-face-small
'Donald Duck thinks that he is stupid.'

b. Donald Duck chèrè nà **yá** / **ọ́** bụ̀ ónyéńzúzù. **Igbo**
Donald Duck think that LogP / OrdP COP stupid.person
'Donald Duck thinks that he is stupid.'

c. Donald Duck rò pé **òún** / **ó** jẹ́ òmùgọ̀. **Yoruba**
Donald Duck think that LogP / OrdP COP stupid.person
'Donald Duck thinks that he is stupid.'

(2) *De se context, say:* (fieldwork)
Elmo goes to visit Big Bird. While there, Big Bird shows him old paintings

[1] Note that we are extending our data set to the matrix verb *promise* in (4) and (8) with additional data points which confirm our hypothesis. These data points are not included in the main paper since we did not investigate strict and sloppy readings of logophors under *promise* as consistently as with the other predicates.

[2] For a way to analyze the cross-linguistic split in *de se* scenarios, see Bimpeh et al. (2024).

he found from back when Elmo was living there with him. After looking at several pictures, Elmo does not recognize one of the paintings which is particularly pretty. Elmo says: "I wonder who painted this. Certainly, the person who painted was a good painter and it is not me, because I'm not very talented in painting!" Later he says "But I did not check! Let me see if I'm the good painter who painted this" Looking carefully at the painting he sees his initials and he says "Oh look there is my signature on it!". Finally he realized.

a. Elmo bé **yè** / **#é** nyé nùtálá nyùìé aɖé. **Ewe**
Elmo say LOGP / ORDP is thing.draw.one-who good INDF
'Elmo said that he is a good painter.'

b. Elmo sị nà **yá** / **ọ́** nà-ésè íhé ǹké ọmá. **Igbo**
Elmo say that LOGP / ORDP IPFV-paint thing nke good
'Elmo said that he is a good painter.'

c. Elmo so pé **òún** / **ó** jẹ́ akunlé tí ó dára. **Yoruba**
Elmo say that LOGP / ORDP is painter REL RP good
'Elmo said that he is a good painter.'

(3) *De se context, hope:* (fieldwork)
Goofy is a candidate in the election. He wants to get elected.

a. Goofy lè mɔ́-kpɔ́-m bé **yè** / **#é** à ɖù àkɔ̀-dádá lá dzí.
Goofy COP path-see-PROG that LOGP / ORDP IRR win vote-cast DEF post
'Goofy hopes that he will win the election.' **Ewe**

b. Goofy nà èlé ánya nà **yá** / **ọ́** gà è-mérí n' àtúḿvóòtù áhù.
Goofy have look eye that LOGP / ORDP FUT PTCP-win in election DEM
'Goofy hopes that he will win the election.' **Igbo**

c. Goofy ń rètí pé **òun** / **ó** yoo borí nínú ìbò náà.
Goofy PROG hope that LOGP / ORDP FUT win in election DET
'Goofy hopes that he will win the election.' **Yoruba**

(4) *De se context, promise:* (fieldwork)
Kofi/Aki/Adé broke his mom's very expensive grinding bowl. He was very sorry. He decided to make it up to her. He made a promise to his mom that he would buy a new one.

a. Kofi doŋugbe be **yè** / **#é** a ƒle ʋe gba yéyê.
Kofi promise that LOGP / ORDP IRR buy earthen ware new
'Kofi promised that he will buy a new grinding bowl.' **Ewe**

b. Ákí kwèrè ńkwà nà **yá** / **ọ́** gà à-zù éféré
Aki promise promise that LogP / OrdP FUT PTCP-buy bowl
ògwùgwé óhùru.
grinding new
'Aki promised that he will buy a new grinding bowl.' **Igbo**

c. Adé se ìlérí wípé **òun** / **ó** maa ra abọ́ ọlọ tuntun.
Ade make promise that LogP / OrdP FUT buy bowl grinder new
'Adé promised that he will buy a new grinding bowl.' **Yoruba**

(5) *De re context, think:* (*Bimpeh et al., 2024*)
Donald Duck (DD) went to the grocery store to buy flour. He mistakenly put sugar in his cart. DD then saw a trail of sugar going up and down the aisles and thought that someone's bag had a hole in it and looked around for the guy. DD says: "I wonder who is losing sugar; certainly, the guy who is losing sugar is stupid, as he does not check his bag". Later he says: "Is it me the stupid guy who is losing sugar?" "No, because I did not buy sugar but flour".

a. Donald Duck súsú bé **#yè** / **é** dzɔ̀mɔ̀ví. **Ewe**
Donald Duck think that LogP / OrdP stupid.person
'Donald Duck thinks that he is stupid.'

b. Donald Duck chèrè nà **#yá** / **ọ́** bụ̀ ónyéńzúzù. **Igbo**
Donald Duck think that LogP / OrdP COP stupid.person
'Donald Duck thinks that he is stupid.'

c. Donald Duck rò pé **#òun** / **ó** jẹ́ òmùgọ̀. **Yoruba**
Donald Duck think that LogP / OrdP COP stupid.person
'Donald Duck thinks that he is stupid.'

(6) *De re context, say:* (*Bimpeh et al., 2024*)
Elmo goes to visit Big Bird. While there, Big Bird shows him old paintings he found from back when Elmo was living there with him. After looking at several pictures, Elmo does not recognize one of the paintings which is particularly pretty. Elmo says: "I wonder who painted this. Certainly, the person who painted is a good painter". Later he says: "Is it me the good painter who painted this? No, because I am not very talented in painting".

a. Elmo bé **#yè** / **é** nyé nùtálá nyùìé aɖé. **Ewe**
Elmo say LogP / OrdP is thing.draw.one-who good INDF
'Elmo said that he is a good painter.'

b. Elmo sị nà **#yá** / **ọ́** nà-ésè íhé ǹké ọ́má. **Igbo**
Elmo say that LogP / OrdP IPFV-paint thing nke good
'Elmo said that he is a good painter.'

c. Elmo so pé **#òún** / **ó** jẹ́ akunlé tí ó dára. **Yoruba**
Elmo say that LogP / OrdP is painter REL RP good
'Elmo said that he is a good painter.'

(7) <u>*De re context, hope:*</u> (fieldwork)
Goofy is so drunk that he has forgotten he is a candidate in the election. He watches someone on TV and finds that that person is a terrific candidate, who should definitely be elected. Unbeknownst to Goofy, the candidate he is watching on TV is Goofy himself.

a. Goofy lè mɔ́-kpɔ́-m bé **#yè** / **é** à ɖù àkɔ̀-dádá
Goofy COP path-see-PROG that LogP / OrdP IRR win vote-cast
lá dzí.
DEF post
'Goofy hopes that he will win the election.' **Ewe**

b. Goofy nà èlé ánya nà **#yá** / **ọ́** gà è-mérí n'
Goofy have look eye that LogP / OrdP FUT PTCP-win in
àtúm̀vóòtù áhù.
election DEM
'Goofy hopes that he will win the election.' **Igbo**

c. Goofy ń rètí pé **#òun** / **ó** yoo borí nínú ìbò náà.
Goofy PROG hope that LogP / OrdP FUT win in election DET
'Goofy hopes that he will win the election.' **Yoruba**

(8) <u>*De re context, promise:*</u> (fieldwork)
Kofi/Aki/Adé threw a party with friends last night at his parents' house. He got so drunk that he broke his mom's expensive grinding bowl. In the afternoon when he woke up, he saw the broken grinding bowl to his horror but totally forgot it was him who broke it. He thought it was someone else. The next day his mom comes home, sees the mess with the grinding bowl and starts yelling at him. Kofi/Aki/Adé tries to calm her down with these words: "I promise you that whoever broke your grinding bowl will pay for a new one."

a. Kofi doŋugbe be **#yè** / **é** a ƒle ʋe gba yéyê.
Kofi promise that LogP / OrdP IRR buy earthen ware new
'Kofi promised that he will buy a new grinding bowl.' **Ewe**

b. Ákí kwè-rè ńkwà nà **#yá** / **ọ́** gà à-zù éféré
Aki promise-PST promise that LogP / OrdP FUT PTCP-buy bowl
ògwùgwé óhùru.
grinding new
'Aki promised that he will buy a new grinding bowl.' **Igbo**

c. Adé se ìlérí wípé **#òun** / **ó** maa ra abọ́ ọlọ
Ade make promise that LOGP / ORDP FUT buy bowl grinder
tuntun.
new
'Adé promised that he will buy a new grinding bowl.' **Yoruba**

We further investigated whether the *de re* reading becomes available if LOGP is focused, following a suggestion in Pearson (2017, 2022). This was based on the observation for English 'he himself' which is often argued to only receive a *de se* reading, though when focused can be read *de re*, see (9).

(9) *Context: Two speakers, A and B. A says:* (Pearson, 2017)
John sees himself in a mirror, but fails to recognize that it's him. He thinks, 'that guy is an idiot', but not 'I am an idiot'.
B says: I don't understand the story. Who does John think is an idiot?
A says: John thinks that he himself is an idiot.

In (10), we present a modified version of the *de re* context (5) and the results for the target sentences in Ewe. We can report that the adjusted scenario did not make the logophor more available. Indeed, the judgements in (10a) and (10b) do not differ from the judgements of the target sentences in (5). Since in Ewe focus is marked morphologically, we also investigated the acceptability of an overtly marked focused logophor in (10c). This sentence too was judged infelicitous.

(10) *Context: Two speakers, A and B. A says:*
Donald Duck (DD) went to the grocery store to buy flour. He mistakenly put sugar in his cart. DD then saw a trail of sugar going up and down the aisles and thought that someone's bag had a hole in it and looked around for the guy. DD says: "I wonder who is losing sugar; certainly, the guy who is losing sugar is stupid, as he does not check his bag". Later he says: "Is it me the stupid guy who is losing sugar?" "No, because I did not buy sugar but flour".
B says:
I don't understand what happened. Who does Donald Duck think is stupid?
A says:

a. #Donald Duck súsú bé **yè** dzɔ̀mòví. **Ewe**
Donald Duck think that LOGP stupid.person
'Donald Duck thinks that he is stupid.'

b. Donald Duck súsú bé **é** dzɔ̀mòví.
Donald Duck think that ORDP stupid.person
'Donald Duck thinks that he is stupid.'

c. #Donald Duck súsú bé **yè-yé** dzɔ̀mòví.
Donald Duck think that LOGP-FOC stupid.person
'Donald Duck thinks that it is he who is stupid.'

We conclude that at least for Ewe focus does not facilitate a *de re* reading of logophors. As for Yoruba and Igbo, testing scenarios like (9) is confounded by the fact that the logophor is form-identical to emphatic pronouns in these languages. In (11), we can observe that the forms *òun* and *yá* can occur in unembedded environments if they are focused, signaled in (11a) with the focus marker in Yoruba, and in (11b) by the presence of a cleft sentence in Igbo.

(11) a. **Òun** ni Tọ́lá rí. **Yoruba**
3SG.EMPH FOC Tola see
'It is her/him/it that Tola saw.' (Manfredi, 1987, 103)

b. Ọ́ wụ́ **ya** kà ḿ hụ̀-rụ. **Igbo**
EXPL be 3SG.EMPH that 1SG see-ASP
'It is her/him/it that I saw.' (Manfredi, 1987, 107-108)

Since the context in (9) is designed to trigger the occurrence of a focused pronoun (due to the added question by speaker B) and focused pronouns are identical in form to LOGP in Yoruba and Igbo, the results of the adjusted scenario would not be very informative. In other words, we would not be able to distinguish focused LOGP from focused ORDP by purely looking at the morphological form since in these dedicated positions the forms are neutralized.

While the fact that some languages display an interaction of focus and logophoricity certainly warrants further investigation, we believe these observations are orthogonal to the availability of *de re* readings of logophors in focus. Rather,

what we should ask is why in some languages the same morphological form realizes LOGP and focused ORDP.[3]

3 Ewe *yè* vs. *yè-a*

Satık (2019, 2020, 2021) identifies *yè-a* in Ewe as the non-finite counterpart of *yè*, where the former is a form that appears under what we cross-linguistically categorize as Obligatory Control (OC) predicates and is essentially *yè* undergoing coalescence with the irrealis marker. The claim is that *yè-a* constitutes overtly spelled out PRO (where the irrealis marker signals non-finiteness), whereas *yè* is a logophor that can appear in finite clauses. Satık provides data in support of this claim wrt. *de re* readings and long distance antecedents: like PRO *yè-a* cannot take long distance antecedents and only allows for *de se* readings, whereas *yè* can take long distance antecedents and can be read *de se* and *de re*. As is evident throughout our paper, our results question Satık's observations and proposal. For example, we provide two mistaken identity scenarios, one for the attitude verb 'hope' requiring *yè-a* in Ewe and one for 'think', which tends to occur with embedded *yè*. Both forms, however, only receive *de se* readings for our consultants. Similarly, in the multiple embedding structures both *yè-a* and *yè* can take long distance antecedents. So at least for our speakers, the irrealis marker does not seem to indicate whether *yè* is a logophor or spells out OC PRO.

Given that the type of attitude predicate consistently varies in Satık's data, in the sense that embedded clauses with *yè* occur with predicates typically selecting finite clauses, and embedded clauses with *yè-a* occur with predicates typically selecting non-finite clauses (at least in English), it is not easy to tell if the behaviour of *yè-a* is different from *yè* due to the irrealis marker or due to the embedding predicate (or both). Fortunately, however, we could observe with our speakers that the irrealis marker *á* can indeed optionally occur under 'say' and 'think', and when it does it

[3]One language where this interaction becomes remarkably transparent is Jula, a Mande language spoken in West Africa, see Kiemtoré (2022) for discussion.

signals future orientation. In (12), we show the effect of the irrealis marker and signal the interpretive effect in the translation.

(12) *The irrealis marker in* ***Ewe*** (fieldwork)

a. Kòkú$_1$ bé **yè**$_{1/*2}$ lɔ̃ Àfí.
Koku say LOG love Afi
'Koku said that he loves Afi.'

b. Kòkú$_1$ bé **yè**$_{1/*2}$ **á** lɔ̃ Àfí.
Koku say LOG IRR love Afi
'Koku said that he will love Afi (one day).'

c. Kòkú$_1$ súsú bé **yè**$_{1/*2}$ lɔ̃ Àfí.
Koku think that LOG love Afi
'Koku thinks that he loves Afi.'

d. Kòkú$_1$ súsú bé **yè**$_{1/*2}$ **á** lɔ̃ Àfí.
Koku think that LOG IRR love Afi
'Koku thinks that he will love Afi (one day).'

Since the contrasts in (12) provide better minimal pairs to investigate the contribution of the irrealis marker isolated from the embedding predicate, we can conduct a small investigation based on the verbs 'say' and 'think'. First, we test long distance antecedents. As is shown in (13), neither combination of 'say' and 'think' leads to an observation where *yè-a* only takes the local attitude holder as an antecedent. If we compare the data in (13) with the multiple embedding structures in the main paper, we find that the addition of the irrealis marker does not restrict *yè* to co-refer only with the local antecedent.

(13) *Long distance antecedents with yè-a in* ***Ewe*** (fieldwork)

a. Kòkú$_1$ súsú bé Kòfí$_2$ bé **yè**$_{1/2}$ **á** ɖè Àfí.
Koku think that Kofi say LOG IRR marry Afi
'Koku thinks that Kofi said that he will marry Afi.'

b. Kòkú$_1$ bé Kòfí$_2$ súsú bé **yè**$_{1/2}$ **á** ɖè Àfí.
Koku say Kofi think that LOG IRR marry Afi
'Koku says that Kofi thinks that he will marry Afi.'

Let us now turn to *de re* readings. First, we have to modify the mistaken identity context for the attitude verb 'say' in (6) with a future orientation to make the occurrence of the irrealis marker felicitous, which is given in (14). As can be seen by the judgements of the target sentences, no effect of the irrealis marker was detected. Importantly, the judgements for LOGP and ORDP are the same in (6) and (14).

(14) <u>*De re context, say, future orientation:*</u> (fieldwork)
Big Bird teaches painting to his friends. In the past, Elmo took one of Big Bird's classes and he painted some paintings. One day, Elmo goes to visit Big Bird. While there, Big Bird shows him one painting that he found in the house, which is particularly pretty. Elmo does not recognize that this is one of the paintings he made when he took Big Bird's class. So he says: "This painting is wonderful. Whoever painted this has a great talent. This person will be a good painter one day."

a.	#Elmo	bé	**yè**	**á**	zù	nùtálá	gã	gbè	áɖé	gbè.
	Elmo	say	LOG	IRR	become	thing.draw.onewho	big	day	INDEF	day

'Elmo says that he will be a great artist one day.'
Comment: Not good because Elmo is speaking about himself.

b.	Elmo	bé	**é**	**á**	zù	nùtálá	gã	gbè	áɖé	gbè.
	Elmo	say	3SG	IRR	become	thing.draw.onewho	big	day	INDEF	day

'Elmo says that he will be a great artist one day.'
Comment: This is good because Elmo is refering to whoever did the painting even though we all know that Elmo is the one who painted it.

We conclude from our small investigation that at least for our Ewe speakers, the irrealis marker by itself does not indicate a non-finite clause, in which *yè* spells out OC PRO, as is proposed in Satık (2020, 2021).[4]

If it is not the irrealis marker that determines the status of what is spelled out by *yè*, what else could cause the different judgements in Satık's data? It is, after all, possible that the differences stem from the fact that Satık's examples often make use of attitude predicates which are different from ours. For example, the mistaken identity scenario Satık tested for *yè-a* included the Ewe verb for 'expect' (Satık, 2021, 7), and the long distance antecedent data were based on the Ewe verbs for 'try' and

[4]The connection between the irrealis marker and non-finiteness is in some sense also critically discussed in Satık's own work (Satık, 2021, 5-6). First, non-finiteness is not so easy to investigate given that Ewe does not display subject-verb agreement or tense marking. Second, the irrealis marker can also appear in matrix clauses.

'want' (Satık, 2021, 8). The predicates tested in our study are 'say', 'think', 'hope', 'promise', and 'want'. While we have shown that the irrealis marker does not indicate an OC profile per se, it is still possible that certain predicates impose additional OC restrictions, which could for example influence the ability of the embedded subject to take a long distance antecedent. While we did not find this restriction with our speakers for 'want', such a restriction under 'want' is documented in Gengbe, a Niger-Congo language closely related to Ewe and spoken in Southern Togo and Benin (Grano and Lotven, 2019, p.484, ex.8). Hence, we do not rule out dialectal differences with certain attitude verbs.

4 Logophors vs. exempt anaphors

The notion of logophoricity has played an important role in analyses of so-called exempt anaphora (Sells 1987; Reinhart and Reuland 1993; Charnavel 2020a,b). In this section, we will briefly discuss to what extent logophoric pronouns in West-African languages and exempt anaphors can be captured with the same analysis.[5] Our preliminary conclusion is that they should not receive the same analysis.

First let us consider some properties that exempt anaphora share with logophoric pronouns. For one, strict readings are attested for exempt anaphors (see Lebeaux 1984, Reinhart and Reuland 1993, Charnavel 2020a). This is shown with VP-ellipsis and English *himself* acting as an exempt anaphor in (15).

(15) John_1 thought there were some pictures of **himself**$_1$ inside, and Bill did, too.
$\rightsquigarrow_{sloppy}$ Bill thought that there were some pictures of himself inside too.
$\rightsquigarrow_{strict}$ Bill thought that there were some pictures of John inside too.
(Lebeaux, 1984, 346)

Moreover, exempt anaphors can only receive *de se* readings, as is exemplified with the Italian exempt anaphor *proprio* in (16) and (17), taken from Chierchia

[5] For the data in this section, we consulted one speaker per language.

(1989). The exempt anaphor is licensed in a *de se* context in (16), but is infelicitous in the mistaken identity context set up in (17).

(16) *Context de se:* Pavarotti sees a man in the mirror with his pants on fire. After a while he realizes for obvious reasons that the man in the mirror is he himself. Pavarotti thinks: "My pants are on fire."

Pavarotti	crede	che	i	**propri**	/	suoi	pantaloni	siano	in	fiamme.
Pavarotti	believes	that	DEF	POSS.SELF	/	POSS	pants	are	on	fire

Pavarotti believes that his pants are on fire.

(17) *Context non de se:* Pavarotti sees a man in the mirror with his pants on fire. The man happens to be Pavarotti himself but he doesn't realize this. Pavarotti thinks: "His pants are on fire."

Pavarotti	crede	che	i	**#propri**	/	suoi	pantaloni	siano	in	fiamme.
Pavarotti	believes	that	DEF	POSS.SELF	/	POSS	pants	are	on	fire

Pavarotti believes that his pants are on fire.

Charnavel (2020a,b) proposes that exempt anaphors should be considered a subcase of local anaphors, thereby capitalizing on the observation that they show identical morphological realization. The exempt status is derived by the assumption that anaphors can be bound by a silent covert pronoun which is introduced by a silent logophoric operator, shown in (18).[6] This pronoun is co-indexed with the antecedent, and thus can trigger strict or sloppy readings. Based on Charnavel and Sportiche (2016), Charnavel develops an analysis in which both pronoun and operator form a clausal projection (notated here LGP) which is merged at the periphery of the binding domain for Condition A (*YP* in (18a)). The operator in (18b) ensures obligatory *de se* readings.

(18) Charnavel (2020b, 9,36)

[6] We discuss the account by Charnavel (2020a,b) here specifically, but note that there are many versions of a silent operator account which have been proposed for logophors in particular (Koopman and Sportiche, 1989; Speas, 2004; Safir, 2004; Adésolá, 2005), but also PRO (Chierchia, 1989), exempt anaphors and logophors (Anand, 2006; Baker and Ikawa, 2024), indexiphors (Deal, 2018) and perspectival anaphora (Sundaresan, 2018).

a. ... DP_i ... $[_{YP}$ $[_{\mathrm{LGP}}$ pro_{log-i} OP_{log} $[_\alpha$ ... ANAPHOR$_i$ ...]]]

b. $[\![\mathrm{OP}_{log}]\!] = \lambda\alpha\lambda x.\alpha$ from x's perspective

While an analysis of logophoric pronouns in Ewe, Yoruba and Igbo in terms of exempt anaphors along the lines of Charnavel's (2020a; 2020b) theory cannot a-priori be ruled out, we think that there are two central problems with it. The first comes from the morphology of logophors. The elegance of Charnavel's theory stems from the fact that exempt anaphors are subsumed under standard local anaphora in languages like English, French, Japanese etc. This is desirable as they share the morphology with local anaphora in these languages. However, at least in Ewe and Yoruba, the reflexive pronoun looks radically different from the logophoric pronoun, shown in (19) and (20).[7] Hence, it is not clear what would motivate a unification of the grammar of LOGPs with that of a exempt anaphors in these languages.

(19) *Reflexives in Ewe* (Clements, 1975)

a. Kòkú kpɔ́ **é-dòkùì** / ***yè**.
Koku saw **3SG-REFL** / **LOGP**
'Koku saw himself.'

b. Kòkú bé yè kpɔ́ yè-dókùì.
Koku say LOG see LOG-REFL
'Koku said that he saw himself.'

(20) *Reflexives in Yoruba* (Lawal, 2006)

a. Taiwo féron **araare**.
Taiwo like **REFL**
'Taiwo likes himself.'

b. *Taiwo féron **óun**.
Taiwo like **LOGP**
'Taiwo likes himself.'

[7] In Igbo, the form of the logophor *yá* is identical to the morphological form of the pronoun in object position. Reflexives like 'herself' are formed by *ònwé* + *yá* (Uchechukwu, 2011). While this could signal that the reflexive and the logophor share a morpheme, it is more likely that the *yá* in *ònwé yá* is simply the pronoun marked for accusative, similar to English *her-self*.

The second problem we see in unifying logophors with exempt anaphors is based on the syntactic and semantic distribution. The licensing conditions of LOGPs appear to be different in complex ways from those of exempt anaphors. Specifically, LOGPs seem to be more narrowly licensed. For example, it is known that an exempt anaphor in English need not necessarily be c-commanded by a suitable antecedent (Ross 1970; Lebeaux 1984, Charnavel 2020a, chapter 1.1), as is shown in (21).

(21) *Exempt anaphor in English*

Mary's anger sometimes turns against **herself**.

In the same position, however, the logophoric pronoun in Ewe is unacceptable (22a). Instead, either the strong 3rd person pronoun (22b) or a reflexive (22c) is used.[8]

(22) *Probing for exempt anaphor in Ewe* (fieldwork)

a. *Kòfí ƒé dzìkú lè ɣèɣíɣí-áɗé-wó-mè trɔ́-ná ɖé **yè** ŋùtɔ́ dzi.
Kofi POSS anger be times-INDEF-PL-inside turn-HAB onto **LOGP** body top
'Kofi's anger sometimes turns against himself.'

b. Kòfí ƒé dzìkú lè ɣèɣíɣí-áɗé-wó-mè trɔ́-ná ɖé **éyà** ŋùtɔ́ dzí.
Kofi POSS anger be times-INDEF-PL-inside turn-HAB onto **3SG** body top
'Kofi's anger sometimes turns against himself.'

c. Kòfí ƒé dzìkú lè ɣèɣíɣí-áɗé-wó-mè trɔ́-ná ɖé **édókùì** dzi.
Kofi POSS anger be times-INDEF-PL-inside turn-HAB onto **REFL** top
'Kofi's anger sometimes turns against himself.'

A similar observation can be made for LOGP in Yoruba. In contrast to the English exempt anaphor, the logophoric pronoun is unacceptable in the same position (23a).

[8] Note that Ewe seems to display both LOGP and and what can presumably be classified as an exempt anaphor in (22c), which strengthens the suspicion that they are not exactly the same creature.

Instead the pronoun is used (which in this example is assimilated to the preceding vowel), see (23b).

(23) *Probing for exempt anaphor in Yoruba* (fieldwork)

a. *ìbúnú u Kọ́lá má kóbá **óun** nígbàmíràm
anger POSS Kola do affect **LOGP** sometimes
'Kola's anger sometimes turns against himself.'

b. ìbúnú u Kọ́lá má kóbá **a** nígbàmíràm
anger POSS Kola do affect **3SG** sometimes
'Kola's anger sometimes turns against himself.'

The distributional differences align with a recent study by Baker and Ikawa (2024), henceforth B&I, in which a comparison is drawn between a West-African type logophor (Ibibio *ímọ̀*) and an exempt anaphor (Japanese *zibun*) across various environments. They encounter several differences between the two types, one is related to distribution. Concretely, they observe that Japanese *zibun*, but not Ibibio *ímọ̀*, is licensed in a root clause if an adjunct like 'according to X' or 'in X's opinion' is present, see (24).

(24) *Logophor vs. exempt anaphor* (Baker and Ikawa, 2024, 900)

a. *Ke akikere Okon, **ímọ̀** i-ma i-due. *Ibibio*
in thought Okon **LOGP** 3.LOG-PST-3.LOG-guilty
'In Okon$_i$'s opinion, Emem/he$_i$ was guilty.'

b. Taroo-ni.yoruto **zibun**-wa waruku-nai-?(n(o)-da-)soo-da. *Japanese*
Taroo-according.to **self**-TOP bad-NEG-no-COP-EVID-COP
'According to Taroo$_i$, self$_i$ is not bad.'

The Ewe logophor *yè* patterns with the logophor in Ibibio and not with the exempt anaphor in Japanese, as the following examples show.[9]

[9]The unacceptability of *yè* in (26b) is not due to the fact that the logophor is accompanied by a focus marker since a focused logophor is in principle possible, see the example in (25), taken from Bimpeh (2023, 162).

(25) Mary be **yè** **yé** dzó.
Mary say **LOGP** **FOC** leave
'Mary said it was she who left.'

(26) *B&I context (24) in Ewe* (fieldwork)

a. Lè Òkón$_1$ ƒé sùsú mè lá, **Kòfí** / **é**$_{1/2}$ / ***yè** xɔ̀sè bé
in Okon POSS mind inside TOP **Kofi** / **3SG** / **LOGP** believe that
yè nyá àgbàlè.
LOG know book
'In Okon's mind, Kofi/he believes he is smart.'

b. Lè Òkón$_1$ ƒé sùsú mè lá, **éyà yé** / ***yè yé** nyé
in Okon POSS mind inside TOP **3SG FOC** / **LOGP FOC** is
àmè sì nyá nú lè Xéxé lá mè.
person REL know thing LOC world DEF inside
'In Okon's mind, he is the smartest person in the world.'

Constructions akin to (26a) produce similar results in Yoruba (27) and Igbo (28). While the ordinary pronoun is licensed, the logophor was not fully accepted by our speakers.

(27) *B&I context (24) in Yoruba* (fieldwork)

a. Ni ọkan Kọ́la, **ó** nigbagbo wipe òun gbon.
in mind Kola **3SG** believe that LOG smart
'In Kola's mind, he believes he is smart.'

b.?? Ni ọkan Kọ́la, **òun** nigbagbo wipe òun gbon.
in mind Kola **LOG** believe that LOG smart
'In Kola's mind, he believes he is smart.'
Comment: It is marked. In fact, speakers might have difficulty parsing it and connecting the reference.

(28) *B&I context (24) in Igbo* (fieldwork)

a. N'uche Okon, **ọ́** kwere ne yá ma ihe.
PREP=mind Okon **3SG** believe that LOG know thing
'In Okon's mind, he believes he is smart.'

b.?? N'uche Okon, **yá** kwere ne yá ma ihe.
PREP=mind Okon **LOG** believe that LOG know thing
'In Kola's mind, he believes he is smart.'
Comment: It sounds degraded to me.

In light of such data, a proposal along the lines of Charnavel (2020a,b) would have to explain why the silent logophoric operator, proposed in (18), which (locally)

binds English/French/Japanese exempt anaphora cannot appear in the same environments to bind the LOGP in Ewe/Yoruba/Igbo. We leave the development of a unifying theory to future work.

References

Adésolá, O. 2005. *Pronouns and Null Operators – A-bar Dependencies and Relations in Yorùbá*. Ph. D. thesis, Rutgers University.

Ameka, F.K. 1991. *Ewe: Its grammatical constructions and illocutionary devices*. Ph. D. thesis, Australian National University Canberra.

Anand, P. 2006. *De de se*. Ph. D. thesis, MIT.

Awde, N. and O. Wambu. 1999. *Igbo-English, English-Igbo Dictionary and Phrasebook*. Hippocrene Books.

Baker, M. and S. Ikawa. 2024. Control Theory and the Relationship between Logophoric Pronouns and Logophoric Uses of Anaphors. *Natural Language and Linguistic Theory* 42: 897–954 .

Bamgboṣe, A. 1966. *A grammar of Yoruba*. Cambridge: Cambridge University Press.

Bimpeh, A.A. 2023. *Logophoricity in Ewe: An Empirical-Semantic assessment of yè*. Ph. D. thesis, Goethe University, Frankfurt.

Bimpeh, A.A., I. Driemel, I. Bassi, and S. Silleresi. 2024. Obligatory de se logophors in Ewe, Yoruba and Igbo: Variation and competition, In *Proceedings of the 40th West Coast Conference on Formal Linguistics*, eds. Lu, J., E. Petersen, A. Zaitsu, and B. Harizanov, 1–10. Somerville, MA: Cascadilla Press.

Capo, H.B. 2010. *A comparative phonology of Gbe*, Volume 14. Walter de Gruyter.

Charnavel, I. 2020a. *Locality and logophoricity: A theory of exempt anaphora*. Oxford: Oxford University Press.

Charnavel, I. 2020b. Logophoricity and Locality: A View from French Anaphors. *Linguistic Inquiry* 51: 671–723 .

Charnavel, I. and D. Sportiche. 2016. Anaphor Binding: What French Inanimate Anaphors Show. *Linguistic Inquiry* 47: 35–87 .

Chierchia, G. 1989. Anaphora and Attitudes de se, In *Semantics and Contextual Expressions*, eds. Bartsch, R., J. van Benthem, and P. van Emde Boas, 1–31. Foris: Dordrecht.

Clements, G. 1975. The Logophoric Pronoun in Ewe: Its Role in Discourse. *Journal of West African Languages* 10: 141–177 .

Deal, A.R. 2018. Indexiphors: Notes on embedded indexicals, shifty agreement, and logophoricity, In *UMOP 40: The Leader of the Pack: A Festschrift in Honor of Peggy Speas*, ed. Ivan, R., 59–86. Amherst: GLSA.

Eberhard, D.M., G.F. Simons, and C.D. Fennig eds. 2020. *Ethnologue: Languages of the World* (23 ed.). Dallas, Texas: SIL International. Online version: www.ethnologue.com.

Emenanjo, N. 2015. *A grammar of contemporary Igbo: Constituents, features and processes*. African Books Collective.

Grano, T. and S. Lotven. 2019. Control of logophoric pronouns in Gengbe. *African linguistics across the disciplines*: 325 .

Kiemtoré, A. 2022. *Issues in Jula complementation: structures, relations and matters of interpretation*. Ph. D. thesis, University of Stuttgart.

Koopman, H. and D. Sportiche. 1989. Pronouns, logical variables, and logophoricity in Abe. *Linguistic Inquiry* 20: 555–588 .

Lawal, N.S. 2006. Yoruba Pronominal Anaphor Òun and the Binding Theory, In *Selected Proceedings of the 35th Annual Conference on African Linguistics*, eds. Mugane, J., J.P. Hutchison, and D.A. Worman, 245–257. Somerville, MA: Cascadilla Proceedings Project.

Lebeaux, D. 1984. Locality and Anaphoric Binding. *The Linguistic Review* 4: 343–363 .

Manfredi, V. 1987. Antilogophoricity as Domain Extension in Igbo and Yoruba. *Niger-Congo Syntax and Semantics* 1: 97–117 .

Pearson, H. 2017. He himself and i. *Snippets* 31: 20–21 .

Pearson, H. 2022. On testing for de se and de re construals across languages. *Semantic Field Methods* 4: 1–22 .

Reinhart, T. and E. Reuland. 1993. Reflexivity. *Linguistic Inquiry 24*(4): 657–720 .

Ross, J.R. 1970. On Declarative Sentences, In *Readings in English Transformational Grammar*, eds. Jacobs, R.A. and P.S. Rosenbaum, 222–272. Waltham, MA: Ginn and Company.

Safir, K. 2004. Person, context and perspective. *Rivista di Linguistica* 16: 107–153 .

Satık, D. 2019. The interpretation of the nonfinite logophoric pronoun in Anlo Ewe. Slides of talk given at TripleA.

Satık, D. 2020. Logophoric pronouns are not (always) inherently logophoric, In *Proceedings of the Fifty-fifth Annual Meeting of the Chicago Linguistic Society*,

ed. Eren, O., 381–396. Chicago: Chicago Linguistic Society.

Satık, D. 2021. Control is not Movement: evidence from overt PRO in Ewe. Unpublished manuscript, February 2021, lingbuzz/004685.

Sells, P. 1987. Aspects of logophoricity. *Linguistic inquiry 18*(3): 445–479 .

Speas, M. 2004. Evidentiality, logophoricity and the syntactic representation of pragmatic features. *Lingua* 114: 255–276 .

Sundaresan, S. 2018. Perspective is syntactic: evidence from anaphora. *Glossa: a journal of general linguistics 3*(1) .

Uchechukwu, C. 2011. The reflexive-reciprocal polysemy in igbo. *Unizik Journal of Arts and Humanities* 12: 200–205 .
